# Impact of Alu repeats on the evolution of human p53 binding sites

**DOI:** 10.1186/1745-6150-6-2

**Published:** 2011-01-06

**Authors:** Feng Cui, Michael V Sirotin, Victor B Zhurkin

**Affiliations:** 1 Laboratory of Cell Biology, National Cancer Institute, NIH, Bethesda, MD 20892, USA

## Abstract

**Background:**

The p53 tumor suppressor protein is involved in a complicated regulatory network, mediating expression of ~1000 human genes. Recent studies have shown that many p53 *in vivo *binding sites (BSs) reside in transposable repeats. The relationship between these BSs and functional p53 response elements (REs) remains unknown, however. We sought to understand whether the p53 REs also reside in transposable elements and particularly in the most-abundant Alu repeats.

**Results:**

We have analyzed ~160 functional p53 REs identified so far and found that 24 of them occur in repeats. More than half of these repeat-associated REs reside in Alu elements. In addition, using a position weight matrix approach, we found ~400,000 potential p53 BSs in Alu elements genome-wide. Importantly, these putative BSs are located in the same regions of Alu repeats as the functional p53 REs - namely, in the vicinity of Boxes A/A' and B of the internal RNA polymerase III promoter. Earlier nucleosome-mapping experiments showed that the Boxes A/A' and B have a different chromatin environment, which is critical for the binding of p53 to DNA. Here, we compare the Alu-residing p53 sites with the corresponding Alu consensus sequences and conclude that the p53 sites likely evolved through two different mechanisms - the sites overlapping with the Boxes A/A' were generated by CG → TG mutations; the other sites apparently pre-existed in the progenitors of several Alu subfamilies, such as AluJo and AluSq. The binding affinity of p53 to the Alu-residing sites generally correlates with the age of Alu subfamilies, so that the strongest sites are embedded in the 'relatively young' Alu repeats.

**Conclusions:**

The primate-specific Alu repeats play an important role in shaping the p53 regulatory network in the context of chromatin. One of the selective factors responsible for the frequent occurrence of Alu repeats in introns may be related to the p53-mediated regulation of Alu transcription, which, in turn, influences expression of the host genes.

**Reviewers:**

This paper was reviewed by Igor B. Rogozin (nominated by Pavel A. Pevzner), Sandor Pongor, and I. King Jordan.

## Background

**Figure 1:**
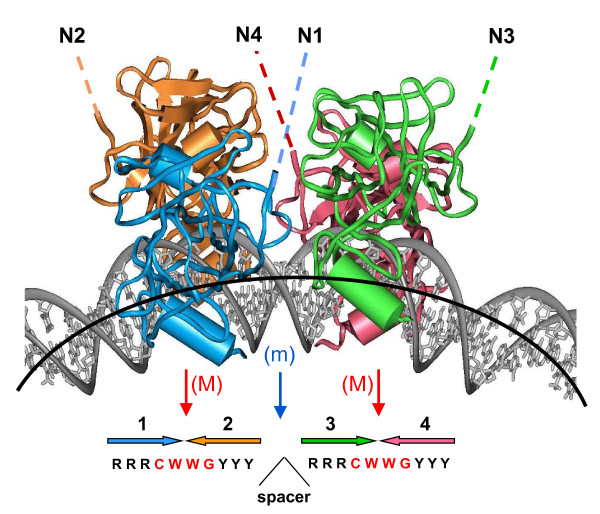
**Four p53 core domains bound to bent DNA**. It is based on the computational model [45] that was further corroborated by gel electrophoresis experiments [46]; the overall DNA bend is ~40°. The spacer S between two decamers varies from 0 to 18 bp in the known functional p53 REs [5,6]. The shown structure corresponds to S = 0. The red arrows show the major-groove bending (M) in the CWWG tetramers; blue arrow is for the minor-groove bend (m) in the center of the site. The lateral positioning of p53 DBDs on the external side of DNA loop and the degree of DNA bending imply that in principle, the p53 tetramer can bind to nucleosomal DNA. The dashed lines indicate that the N-termini N1-N4 are accessible for interactions with trans-activation and trans-repression factors (*e.g*., histone acetylase, HAT, and histone deacetylase, HDAC). Large colored arrows indicate the orientations of the four p53 subunits.

P53 is one of the best-known tumor suppressor proteins, and is involved in an amazingly complicated regulatory network [1-3]. In response to various kinds of cellular stress, p53 induces activation and repression of more than a thousand human genes [4]. The p53 protein possesses the classical features of a eukaryotic transcription factor, including a sequence-specific DNA binding domain (DBD), as well as transactivation and tetramerization domains. Upon activation, the p53 tetramer binds to DNA sequence-specifically, but this specificity is extremely degenerate. A typical p53 binding site (BS) comprises two decamers RRRCWWGYYY separated by a variable spacer, S [5]; see Figure 1. (Here, R is purine; Y is pyrimidine; W is A or T.) The strongest p53 sites, such as *p21*, have spacer S = 0, but spacers as long as S = 18 bp can also be functional [6]. As a consequence of this degeneracy, the human genome contains enormous numbers of potential p53 BSs. The functional significance of the vast majority of these sites remains unknown, however.

Experimentally, the p53 BSs have been identified by two approaches: one is a traditional, single-gene approach, and the other is a genome-wide approach. The first approach [6] focuses on a specific target gene, usually including a transcription assay that uses a reporter-gene construct to determine the specific DNA fragment interacting with p53. (The latter is denoted p53 response element, RE). The second approach [7,8] is based on cross-linking p53 to genomic DNA, with subsequent extraction of the p53-bound DNA fragments by chromatin immuno-precipitation (ChIP). Although some p53 ChIP fragments overlap with previously established p53 REs such as the *p21 *5'-site, most of them remain uncharacterized [7]. One critical distinction between these two sets of p53 sites lies in their positioning with respect to the transcription start sites (TSS) of the nearest genes: while ~70% of p53 REs are located within 2 kb from TSS, less than 10% of p53 ChIP fragments are within this range, indicating that physically they reside in different genomic regions (Additional File 1: Table S1). Below, we distinguish between the functional p53 REs proven to be critical for regulation of expression of the corresponding genes, and the p53 ChIP fragments and predicted p53 BSs, whose function is mostly unknown.

While the p53-induced activation of transcription has been studied the most extensively, more than 50% of p53-modulated genes are repressed by p53, and this fraction may actually be as high as 80% [9]. However, the most comprehensive collection published recently contains ~160 REs for ~130 genes, almost all of which are activated by p53 [6]. Therefore, our knowledge of the mechanisms of p53-mediated trans-repression still remains very limited. According to the current paradigm, the principal difference between trans-activation and trans-repression is explained by the alteration of chromatin structure at target gene promoters through the p53-induced recruitment of co-activators or co-repressors, such as histone acetylase, HAT, and histone deacetylase, HDAC [6,10,11].

To test the hypothesis that the spacer S between two p53 half-sites is related to the p53-mediated trans-activation and repression [6,11], we analyzed *in silico *the putative p53 BSs in the promoters of genes regulated by p53 [4]. The stereochemical rationale for this hypothesis was that the spacer length determines the relative orientation between the two half-sites and between the two p53 dimers bound to DNA (Figure 1); this, in turn, determines which co-factors would be recruited to the p53-DNA complex. We found that the genes which are up- and down-regulated by p53 do indeed differ in the spacer length: S = 0 is predominant for the up-regulated genes, while S = 3 bp is over-represented for the down-regulated genes [4] (M.V. Sirotin and V.B. Zhurkin, unpublished observation). These findings are consistent with the known data for two p53-trans-repressed genes, *MAP4 *and *survivin*, whose p53 REs contain a 3-bp spacer. Furthermore, deletion of this spacer (that is, changing the spacer S = 3 to S = 0) converts the *survivin *p53 site into a trans-activating element [11].

Initially, we found that a significant fraction of the predicted p53 BSs in the human genome is organized in multiple tandem repeats and some of these sites are embedded in the LTR-transposons of THE1-MaLR family [12]. Later, the list of transposon families containing the putative p53 sites was extended to include SINE/Alu, SINE/MIR and LTR/ERV [13]. These results were substantiated by Haussler and coworkers [14] who analyzed numerous p53 BSs detected in the p53-ChIP experiments [7] and showed that ~1500 of these sites are embedded in ERV LTR regions. Moreover, the distributions of the length of the spacer, S, derived from human and mouse genomes proved to be different [15], suggesting that the primate-specific interspersed repeats may contribute to the observed differences. Therefore, in this study we further expand this analysis, with the most attention directed to the primate-specific Alu repeats.

More than one million Alu sequences are scattered throughout the human genome, representing ~10% of its length [16]. Recently, it became clear that Alu and other transposable elements are indispensable for the evolution of regulatory networks [17-19]. This idea was first put forward by Britten and Davidson [20]. Later, numerous Alu elements were found in promoters and enhancers of genes, suggesting that Alu elements may function as carriers of *cis *regulatory elements modulating gene expression [21-30]. In particular, various transcription factor BSs have been established in Alu elements, including GATA [31], LyF-1 [31], Sp1 [32,33], YY1 [33,34] and retinoic acid receptors (RARs) [35].

An important observation was made recently by Vingron and coworkers [36], who found that a substantial number of p53 sites detected in cross-linking experiments (p53 ChIP fragments) reside in Alu elements. Comparing the sequences of the p53 sites with their counterparts in the Alu consensus sequences, the authors detected multiple CG dinucleotides occurring at the positions that correspond to the CATG tetramer in the p53 sites. They therefore proposed that the methylation and deamination of cytosine that results in the CG → TG transition could generate the CATG motifs attractive to p53 for binding *in vivo*. (Independently, we proposed the same mechanism for thousands of predicted p53 BSs residing in Alu repeats genome-wide [15].) This mechanism, however, ostensibly differs from the one proposed earlier for the ERV LTR families by Haussler and coauthors [14], who argued that the p53 BSs are likely present in progenitor LTRs, not generated through mutations. Note also that the cited studies did not address the question whether the Alu elements and other repeats harbor functional p53 REs directly involved in regulation of transcription.

To clarify these issues, we analyzed the two 'extreme' datasets, functional p53 REs and putative p53 BSs. The first set contains a relatively small number (~160) of rigorously defined p53 REs [6], while the second set of p53 sites, predicted using the position weight matrix approach PWM-20 (see Methods), includes ~2 million putative p53 sites in the human genome. We found that out of the ~160 p53 REs, 24 REs occur in repetitive DNA, 13 of them residing in Alu repeats. These thirteen p53 REs are clustered in the three 'hot-spot' regions in Alu repeats, overlapping with the Boxes A/A' and B of the internal RNA polymerase III (pol III) promoters. Importantly, numerous putative p53 BSs are also clustered in the same three regions in Alu repeats. A comparison of the sequences of p53 sites and their Alu counterparts revealed that Alu-residing p53 sites probably evolved through two different mechanisms: the sites overlapping with Boxes A/A' were generated by CG → TG mutations; the sites overlapping with Box B apparently pre-existed in the progenitors of the corresponding Alu subfamilies. This indicates that the two mechanisms for generation of p53 sites proposed earlier [14,36] may both be operative in the human genome.

Finally, we examined the three 'hot spots' in the context of nucleosome positions in Alu repeats [37,38] and found that the p53 sites near Box B are located in the nucleosome-free linker region, while the sites overlapping with Boxes A/A' are covered by nucleosomes. Further analysis of the DNA rotational orientation showed that the latter sites are positioned in nucleosomes in such a way that they are 'exposed' on the nucleosome surface, facilitating p53 binding [39]. Since the upstream regions of the TSS (several kilobases in length) are enriched with Alu elements [40], it is conceivable that p53 may utilize at least some of the predicted BSs to modulate transcription of certain groups of the human genes in response to cellular stresses.

## Results

### Functional human p53 REs residing in repeats

Analysis of the known p53 sites and their flanking genome fragments (157 in total, see Methods) revealed 24 functional sites that are located in repeats (Table 1). These repeats belong to various families, including SINE/Alu, SINE/MIR, LTR/ERV, consistent with our earlier predictions [12,13] and the results of analysis of the p53 ChIP fragments obtained *in vivo *[14,36]. Among these repeat-associated p53 REs there are several well-established sites modulating cell cycle arrest genes, such as CCNK [41], and apoptotic genes, such as AIFM2 [42] and TP53INP1 [43]. These results confirm the idea that various repeat families might function as a supply of p53 BSs *in vivo *[19].

**Table 1:** Functional human p53 response elements occurring in repeats

**#**	**Gene name^a^**	**RE sequence**	**Spacer, bp**	**Repeat Assignment (CENSOR/RepeatMasker)^c^**	**Class/Family**
1	GDF15	CATCTTGCCC AGACTTGTCT	0	FLAM_C	SINE/Alu
2	BCL2L14	AGCCAAGGCT GGTCTTGAAC	0	AluJr/Jo	SINE/Alu
3	CASP10, RE1	GGGCATGGTG GGACATGCCT	0	AluJo/Jr	SINE/Alu
4	CASP10, RE2	GGGCATGGTG GCACATGCCT	0	AluSp	SINE/Alu
5	BID	GGGCATGATG GTGCATGCCT	0	AluSg	SINE/Alu
6	TSC2, RE1	GGGCATGGTG GCACATGCCT	0	AluSg	SINE/Alu
7	EphA2	AGACATGCCT(S)CAACATGGTG	3	AluSz	SINE/Alu
8	HTT (HD)	CGCCATGTTG(S)AGGCTGGTCT	3	AluSq2	SINE/Alu
9	CASP6	AGGCAAGGAG(S)AGACAAGTCT	4	AluJr	SINE/Alu
10	BNIP3L	AAGCTAGTCT(S)GCGCATGCCT	5	AluJo/Jr	SINE/Alu
11	AIFM2, RE1	AGACCAGCCT(S)TAGCGAGACC	8	AluJo	SINE/Alu
12	AIFM2, RE2^b^	GGGCATGGCC(S)GCTCATGCCT	10	AluJo	SINE/Alu
13	TSC2, RE2	AGGCTAGTCT(S)TGACGTGACC	13	AluJb	SINE/Alu
					
14	CCNK^b^	AAACTAGCTT(S)AGACATGCTG	2	MIRb	SINE/MIR
15	CASP10, RE3	AAACTTGCTG(S)AATCTTGGCT	5	MIR	SINE/MIR
					
16	CTSD^b^	AACCTTGGTT TGCAAGAGGC	0	MER4D	LTR/ERV1
17	MMP2	AGACAAGCCT GAACTTGTCT	0	LTR88b	LTR/Gypsy
18	SCN3B	TGACTTGCTC TGCCTTGCCT	0	THE1B	LTR/ERVL-MaLR
19	TP53INP1	GAACTTGGGG GAACATGTTT	0	MER21B	LTR/ERVL
20	TRIM22	TGACATGTCT AGGCATGTAG	0	LTR10D	LTR/ERV1
21	CRYZ	CTGCAAGTCC(S)AAACCTGTTT	3	THE1B	LTR/ERVL-MaLR
22	PLK2	GGTCATGATT(S)TAACTTGCCT	3	MER34C	LTR/ERV1
					
23	COL18A1	TGACATGTGT GAGCATGTAT	0	(TG)n	Simple_repeat
24	SCARA3	GGGCAAGCCC AGACAAGTTG	0	MER81	DNA/hAT-Blackjac

^a ^In those cases when genes have several REs, they are denoted RE1, RE2, RE3.

^b ^The REs are partially covered (> 5 nt) by repeats.

^c ^CENSOR [61] and RepeatMasker (A.F.A. Smit, R. Hubley, and P. Green, http://www.repeatmasker.org) are used to assign repeats to subfamilies: two assignments are shown if their predictions are different, whereas only one consensus assignment is shown if their predictions are the same.

Note that ~55% of all p53 REs residing in repeats (13 out of 24 REs) are associated with the Alu family (Table 1). Given that Alu repeats comprise ~25% of all repeats in human genome, it is reasonable to assume that Alu elements in general are characterized by a higher 'density' of p53 sites compared to the other repeats (see below).

### Localization of functional p53 REs in Alu repeats

**Figure 2:**
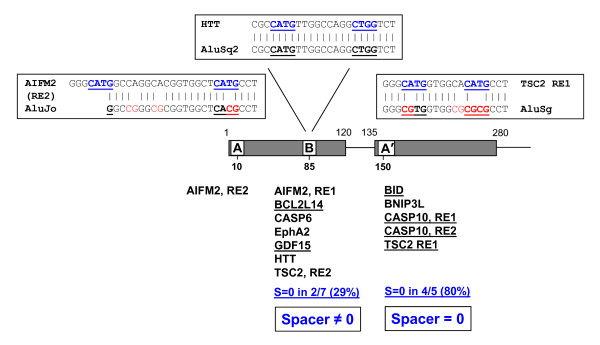
**Mapping functional p53 sites on Alu repeats**. The left and right Alu monomers are represented by rectangles; the inter-monomer region and the 3'-tail are shown by lines. Thirteen p53 REs residing in Alu repeats (Table 1) are localized around positions 10, 85 and 150 overlapping with Boxes A, B and A'. The p53 REs are listed according to their localization in Alu, and the ones with spacer S = 0 are underlined. Alignments of selected p53 REs with the corresponding Alu subfamily consensus sequences are shown. The p53 core motifs CNNG and the corresponding regions in Alu elements are highlighted in boldface. The CG dinucleotides are shown in red. Note that the two p53 REs associated with the AIFM2 gene are localized in the same repeat AluJo (Additional File 3: Figure S2) and separated by one superhelical turn of DNA in nucleosome. In such a case, two p53 tetramers can bind cooperatively on the same side of the nucleosomal surface [12], thereby increasing their affinity to DNA. Simultaneous mutations in Boxes A and B (producing two p53 half-sites in the same Alu repeat, separated by S = 75 bp) are likely to happen relatively often. This can explain an unusually frequent occurrence of the spacer S = 75 bp observed earlier [12].

All p53 REs associated with the Alu subfamilies (Table 1) are mapped to the three locations (or 'hot spots') in Alu repeats, around positions 10, 85 and 150 (Figure 2). These REs can be divided in two groups, A and B, depending on their proximity to the Boxes A/A' or B of the pol III promoters on the left and right monomers of the Alu element. The hot spots at positions 10 and 150 were identified previously by Zemojtel *et al*. [36] in the analysis of *in vivo *p53 BSs. These authors compared sequences of the Alu-residing p53 sites to their counterparts in repeats and found that the latter were enriched with the CG dimers, especially in the CNNG core motifs. Based on this observation it was suggested [36] that methylation and deamination of CpG could generate strong motifs CATG for p53 binding *in vivo*.

Our data are consistent with these results: the Alu regions aligned to the p53 REs from the group A contain numerous CG dinucleotides (Figure 2 and Additional File 2: Figure S1). For example, in the case of TSC2 RE1, the consensus AluSg sequence contains four CG dimers, all of which are substituted by CA:TG dimers in the p53 RE. (The large-scale alignment between the AluSg consensus and the TSC2 RE1 flanked by genomic sequences, leaves practically no doubt that the Alu repeat was indeed a progenitor of this functional p53 site - the 'divergence' ratio (div) is a mere 10.5% for the 290 bp-long DNA fragments (Additional File 3: Figure S2). For the thirteen p53 REs aligned to Alu repeats, this ratio varies from 8.7 to 18.1%, in most cases being less than 15%.)

Note that the CG dimers have been frequently substituted not only in the CNNG tetramers, as was emphasized by Zemojtel *et al*. [36], but rather in the whole ~20 bp-long Alu regions corresponding to the p53 sites (Figure 2 and Additional File 2: Figure S1). In general, however, our analysis of the p53 REs confirms the observations made earlier [36] and provides an additional evidence that the CG → TG transition was a driving force in creating functional p53 sites embedded in Alu repeats at hot spots 10 and 150 (Boxes A/A' in Figure 2).

The p53 REs mapped to Alu repeats around position 85 (Box B in Figure 2) comprise a novel group of p53 sites (denoted as the group B), which differ from the sites described above in several aspects. First, the average density of CG dinucleotides and the number of apparent CG → TG transitions is much lower in the group B (Additional File 2: Figure S1). In particular, comparison of the CNNG core motifs in the p53 sites and in their Alu counterparts shows that there is only one CG → TG transition in the seven REs from group B (BCL2L14 in Additional File 2: Figure S1). By contrast, for the six REs in group A, there are 16 such transitions (the corresponding CG dimers are highlighted in magenta in Additional File 2: Figure S1).

Second, the total number of mutations distinguishing the p53 REs from their Alu precursors is also smaller for the group B. The mutation rate for the group B is nearly twice as small as that for the group A - 0.14 and 0.23 substitutions per base pair, respectively (the numbers of substitutions for each p53 site are given in Additional File 2: Figure S1). For example, in the p53 site associated with the Huntingtin gene, HTT, not only the core motifs CATG and CTGG but also flanking sequences are identical to those in the AluSq2 consensus sequence (Figure 2), suggesting that for a substantial number of the AluSq2 elements interspersed throughout human genome, the Box B regions could serve as the 'natural born' sites for p53 binding.

Third, the two groups of p53 REs differ in the length of spacer, S. In particular, 80% of the REs mapped at position 150 (Box A') have spacer S = 0, whereas for position 85 (Box B), the fraction of p53 sites with spacer S = 0 is less than 30% (Figure 2, Table 1).

In summary, the functional p53 REs mapped to Alu elements can be separated into two groups, characterized by distinctive locations in Alu repeats, the density and mutation rate of CG dimers in the corresponding Alu regions and the spacer length S. This assessment based on a limited set of p53 REs is corroborated by a genome-wide analysis of putative p53 BSs in Alu elements described in the following sections.

### *In silico *identification of putative p53 sites in human genome

**Figure 3:**
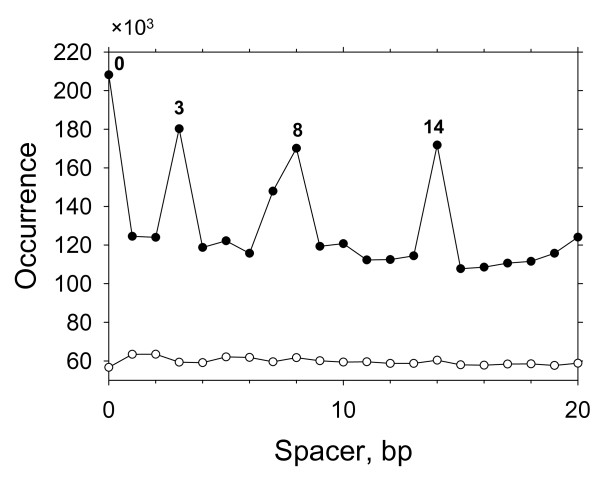
**Occurrence of putative p53 BSs with various spacers S found in unmasked (filled circles) and masked (open circles) human genome**. The prevalent spacer lengths are indicated.

First, we searched for putative p53 BSs in masked and unmasked human genome (NCBI Build 36) to understand how these sites are distributed between the repeat and non-repeat regions. A scan of the unmasked genome found ~2 million p53 sites with the PWM-20 scores of 70% or higher (see Methods). Remarkably, distribution of the spacer lengths in these sites is highly non-uniform, with the peaks at S = 0, 3, 8 and 14 bp (Figure 3). The highest occurrence of ~210,000 sites is observed for spacer S = 0, whereas the background level is ~120,000 sites. The prevalence of the mentioned spacers disappeared, however, when the masked genome was used - in this case, the occurrences of various spacers became nearly equal, ~60,000 sites for each spacer size (Figure 3). This result implies that the p53 sites with selected spacer sizes mentioned above (S = 0, 3, 8 and 14 bp) are overrepresented in repeats. Overall, the repeat regions of human genome contain ~1.05 million potential p53 sites satisfying the criteria listed in Methods (data not shown).

### Distribution of p53 sites in Alu repeats

**Figure 4:**
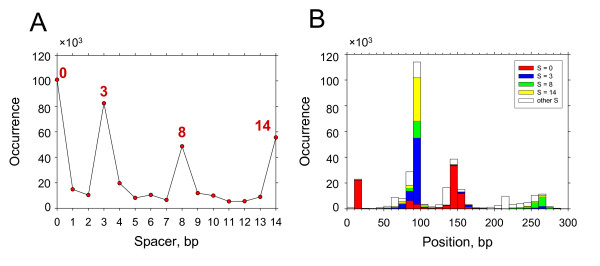
**Localization of putative p53 BSs in Alu repeats**. (A) Occurrence of p53 BSs with spacer S = 0-14 bp in Alu repeats. The prevalent spacers are indicated. (B) Locations of the predicted p53 BSs on Alu repeats. The sites with spacer S = 0, 3, 8 and 14 bp are represented by red, blue, green and yellow bars, respectively. The sites with other spacer lengths are shown by white bars.

A search of putative p53 BSs in Alu repeats using PWM-20 rendered ~0.4 million BSs (Table 2), or 38% of the sites found in all repeats. Therefore, consistent with our findings for the functional p53 REs (see above), the Alu elements have a (somewhat) higher 'density' of putative p53 sites compared to other repeats. Analysis of the spacer lengths in the Alu-residing p53 sites also revealed a non-uniform distribution (Figure 4A): by analogy with Figure 3, majority of the sites have spacers S = 0, 3, 8 and 14 bp. Comparison between the amplitudes of the peaks in Figures 3 and 4A indicates that Alu repeats harbor ~60% of all the sites with these selected spacers, residing in repeats.

**Table 2:** Summary of p53 motifs found in selected Alu subfamilies

Name^a^	# Alu elements^b^	# p53 motifs^c^	S = 0^d^	S = 3^d^	S = 8^d^	S = 14
FLAM-A	18264	5484	633	233	**2080**	1057
FLAM-C	35888	14083	1786	804	**5036**	3923
AluJo	137954	75938	13745	4424	**20989**	14007
AluJb	126856	62528	**12542**	**11954**	4297	9560
AluSx	333710	129530	32361	**42879**	8104	16192
AluSq	93874	30593	7253	**12164**	409	4445
AluSg	84797	30561	**9593**	6280	3608	3136
AluSp	50857	21429	**13340**	1904	127	2036
AluSc	43135	6884	**2945**	864	293	92
AluSg1	6130	4429	**3570**	72	186	200
AluY^e^	139813	12488	1753	512	2542	360

						

Total	1071278	393947	99521	82090	47671	55008

^a ^The Alu subfamilies are ordered by age [80-82,73]. Only subfamilies with the number of p53 motifs exceeding 4000 are presented.

^b ^The Alu elements are collected from AluGene database [65].

^c ^The p53 motifs with the spacer, S = 0-14 bp, and with PWM-20 scores ≥ 70% are counted.

^d ^The numbers of p53 motifs with spacer S = 0, 3, 8, 14 bp are presented. For each subfamily (except AluY), the data corresponding to the prevalent spacers S are highlighted in boldface and underlined.

^e ^For the AluY subfamily, the prevalent spacer is S = 2 with 4588 p53 motifs (Additional File 4: Figure S4-I).

Next, we analyzed positions of potential p53 BSs in Alu elements (Figure 4B) and found that their distribution resembles the one derived from the functional p53 REs (Figure 2). First, note that the p53 sites in Figure 4B are clustered around the same three positions, 10, 85 and 150, as the three hot spots shown in Figure 2. Second, the p53 BSs with S = 0 are distributed differently from those with S ≠ 0. The putative sites with S = 0 occur predominantly around positions 10 and 150 (Figure 4B), coinciding with the two hot spots representing group A; similarly, most of the functional REs positioned here have spacer S = 0 (Figure 2). By contrast, most of the p53 BSs with S ≠ 0 are found near position 85 (Figure 4B), overlapping with the hot spot representing group B (Figure 2). Note that ~70% of the functional REs in group B also have spacer S ≠ 0.

### Spacer distribution in Alu-residing p53 sites correlates with the age of Alu elements

**Figure 5:**
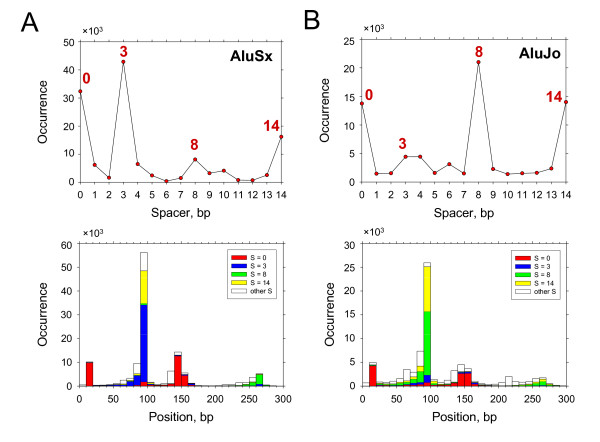
**Occurrence of putative p53 sites in the subfamilies AluSx (A) and AluJo (B)**. The notations are as in Figure 4.

Further analysis of p53 sites associated with Alu repeats revealed a strong variability of the Alu subfamilies in terms of positioning (and distribution) of p53 BSs with different spacers (Figure 5 and Additional File 4: Figure S4). Specifically, for the AluSx elements, the most prevalent spacer is S = 3 (Figure 5A), while for the AluJo elements, the spacer S = 8 is predominant (Figure 5B). Comparison of the most frequent spacer sizes in different Alu subfamilies reveals an interesting tendency - the spacer length, S, generally correlates with the age of Alu elements (Table 2). For example, the 'old' Alu subfamilies FLAM-A, FLAM-C and AluJo are characterized with spacer S = 8; in the 'intermediate' subfamilies AluJb, AluSx, AluSq the spacer is S = 3, while the 'relatively young' subfamilies AluSg, AluSp, AluSc and AluSg1 have the shortest possible spacer S = 0. From the evolutionary standpoint, this trend can be interpreted as an increase in the p53 binding affinity in the younger Alu elements (because the p53 tetramer is known to bind stronger to the sites with shorter spacers [5,6]).

**Figure 6:**
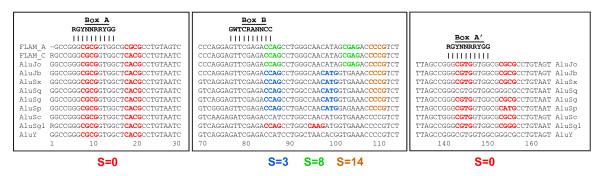
**Localization of putative p53 sites with selected spacer lengths in the Alu elements**. The scheme summarizes the data presented in Figures 5 and S4 (Additional File 4). The consensus sequences of 11 Alu subfamilies were downloaded from Repbase Update [74-76] and aligned using the ClustalW module in BioEdit [77]. Top: The consensus sequences of the Boxes A, B and A' [78] are aligned with the Alu sequences. The CNNG core motifs shown in color indicate predominant positions in the Alu elements where the p53 sites were found. For each Alu subfamily, the distances between the CNNG cores in the consensus sequence are directly related to the predominant spacer lengths observed in the p53 sites residing in the corresponding Alu repeat (Figures 5 and Additional File 4: Figure S4). In the case of AluY subfamily, most of the p53 sites are located around position 215 and have spacer S = 2 (Additional File 4: Figure S4-I). The color code corresponds to the spacer length: red for S = 0, blue for S = 3, green for S = 8 and yellow for S = 14 bp.

Note that in those subfamilies where the predominant spacer is S = 3 or 8, the p53 sites are mostly located near position 85 (Box B, Figure 6 and Additional File 4: Figure S4), which is entirely consistent with the global picture shown in Figure 4B. When the most frequent spacer is S = 0, the corresponding p53 sites are distributed between all the three hot spots 10, 85 and 150 - see AluSg, AluSp, AluSc and AluSg1 in Figure 6 and Additional File 4: Figure S4. (In the subfamilies AluSp and AluSc the p53 BSs with zero spacer length occur at positions 10 and 150; in AluSg these p53 BSs are found at positions 10, 85 and 150; in AluSg1 nearly all p53 BSs are located at position 85.)

The exception is the youngest subfamily AluY with predominant spacer S = 2 and the p53 sites located mostly around position 215 (Additional File 4: Figure S4-I). However, all these families with spacer S = 0 or 2 contain relatively small number of putative p53 BSs (Table 2) and these BSs do not change the general statistics presented in Figure 4.

### Distinctive chromatin context of putative p53 sites embedded in Alu repeats

**Figure 7:**
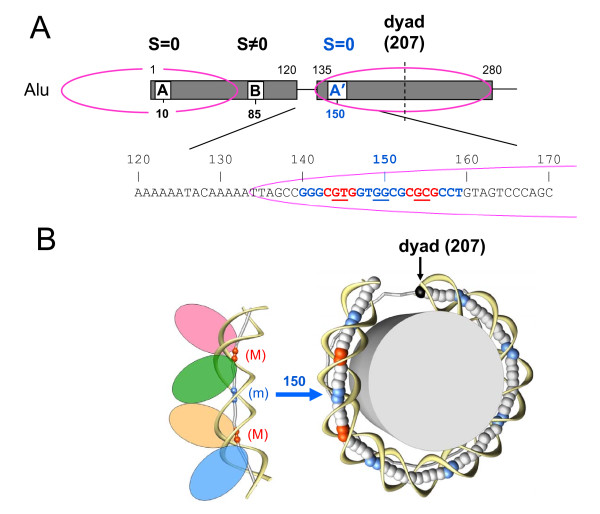
**Experimentally mapped nucleosomes and predicted p53 BSs in Alu elements**. (A) The two mapped nucleosomes (magenta ellipses) are shown together with the three clusters of p53 sites near positions 10 (Box A), 85 (Box B) and 150 (Box A'). For each cluster, the prevalent spacer S is indicated (Figures 2 and 4). The fragment of Alu consensus sequence is presented (positions 120 to 170), with the 20 bp-long counterpart of the predicted p53 site colored in red and blue. The underlined dimers correspond to the base pairs shown as the red and blue balls in the left part of Figure 7B. The approximate *in vitro *nucleosome position in the right Alu monomer has been established by Englander and Howard [37]. The shown dyad position 207 corresponds to the optimal rotational setting of this nucleosome - see main text. (B) Exposure of the p53 site embedded in the nucleosome mapped on the right Alu monomer (covering the interval from 134 to 280). Right: The histone octamer is shown as a cylinder and DNA is represented by ribbons (sugar-phosphate backbone) and balls (centers of base pairs). The blue balls indicate the dimeric steps where DNA is bent into the minor groove [79]. For the 'posterior' half of nucleosome, the DNA axis is represented by grey sticks. Left: The p53-DNA complex (Figure 1) is shown schematically with four ellipses representing the p53 tetramer bound to a 20-bp DNA fragment (spacer S = 0). The DNA axis is represented by sticks and balls. The red balls stand for the centers of the CNNG core motifs bent into the major groove, and the blue ones for the junction between two half-sites bent into the minor groove (underlined in Figure 7A). Note that conformation of the DNA fragment bound by p53 tetramer (on the left) closely resembles conformation of the 20 bp-long fragment of nucleosomal DNA centered at position 150 (on the right). Therefore, the p53 site embedded in nucleosome at this position is likely to be accessible for p53 binding. Centers of the other accessible sites are shown by blue balls.

Earlier, Howard and his colleagues [37,38] found that Alu elements can harbor two nucleosomes: one resides within the right Alu monomer, between the inter-monomer A/T-rich spacer and 3' poly(dA) tract (Figure 7A); the other nucleosome partially overlaps with the left monomer so that its center is located around the 5' end of the Alu repeat. Recent analysis of the genome-wide nucleosome occurrences confirmed this observation [44]. As a consequence, the p53 BSs clustered around position 85 (and characterized by spacer S ≠ 0) are located in the nucleosome-free linker region (Figure 7A). Therefore, they are likely to be easily accessible for p53 binding.

By contrast, the p53 BSs with spacer S = 0 mapped at positions 10 and 150 are embedded in nucleosomes (Figure 7A). In this case, the p53 binding to its cognate site would depend on the rotational orientation of the site with regard to the histone octamer. Based on the results of stereochemical analysis and gel electrophoresis data, we predicted that if the p53 site is properly exposed on the nucleosomal surface and bent into the minor groove in the center of 20-meric motif (Figures 1 and 7B), the p53 tetramer would have a high affinity to such site [45,46]. If, however, the p53 site is positioned in nucleosome in the opposite rotational orientation, the p53 binding would be highly unfavorable (practically impossible without unraveling nucleosomal DNA) due to steric hindrances imposed by the core histones. Recent experiments confirmed this prediction [39].

We are particularly interested in analyzing rotational orientation of the p53 BSs mapped to position 150 and embedded in the 'right' nucleosome (Figure 7A). This nucleosome covers the right Alu monomer and is localized between two long A-tracts at positions ~120/~135 and ~280/~290. Thus, its location is strongly restricted and (almost) does not depend on the neighboring genomic DNA. In other words, the 'right' nucleosome detected by Englander and Howard [37] in the AFP-Alu element and similarly positioned nucleosomes in other Alu repeats are probably the most representative in human genome, covering ~5% of all DNA (because the Alu repeats comprise ~10% of genome, and the 'right' nucleosome covers one half of Alu repeat).

To determine the most favorable rotational positioning of the 'right' nucleosome, we used a theoretical approach [47] based on the well-known sequence pattern in nucleosomal DNA - the AT-rich dimers preferentially occur at the sites of DNA bending into the minor groove, while the GC-rich fragments occur at the sites where DNA is bent toward the major groove. (For details see Set 1 in Table SIII [47]). We found that the rotational orientation of DNA with regard to the histone octamer is optimal when the nucleosome dyad is located at position 207 - that is, just in the center of the 147-bp fragment (positions 134 to 280) between two A-tracts (Figure 7A).

In this case, DNA in position 150 is bent into the minor groove and the p53 site with its center in this position has a conformation remarkably similar to that induced by p53 binding (Figure 7B). In particular, the two core CNNG tetramers are bent into the major groove (the red balls in Figure 7B), whereas the junction between two half-sites is bent into the minor groove (the blue balls in Figure 7B). The strong resemblance between these two DNA conformations suggests that the p53 site embedded in nucleosome can be accessible for p53 binding. This assertion is consistent with recent experiments indicating that p53 tetramer effectively binds its cognate site wrapped in nucleosome if the site is properly exposed on the nucleosomal surface [39].

Based on these observations, we conclude that the two groups of potential p53 sites residing in Alu elements are likely to have distinctive chromatin environment. The sites with spacer S ≠ 0 (position 85) are located in the inter-nucleosome linker regions, while the sites with spacer S = 0 (position 150) are exposed on nucleosomes. In both cases, the p53 sites are expected to be accessible for p53 binding.

## Discussion

### Significant number of putative p53 sites in human genome

The number of p53 binding sites in human genome has been estimated based on experimental data. For example, 37 sites were identified in 1% of human genome (ENCODE) using ChIP assays, which can be extrapolated to ~3,700 sites genome-wide [48]. Analysis of the ChIP assay on human chromosomes 21 and 22 (~3% of genome) revealed 48 binding sites, which can be extrapolated to ~1,500 sites in the whole genome [49]. Two recent genome-wide ChIP experiments detected ~65,000 low-confidence p53 sites [7] and ~1,500 high-confidence sites [8].

The studies using various bioinformatics approaches found the larger numbers of p53 sites. In particular, the algorithm based on the p53 affinity for DNA binding sites predicted over 10,000 high-affinity sites and over 200,000 weaker ones with spacer S = 0-1 [50]. A genome-wide search of the p53 binding motifs consisting of two half-sites RRRCWWGYYY with spacer S = 0 found ~160,000 sites [36]. Using a standard PWM-based approach that allows the spacer S to vary from 0 to 14 bp (see Methods), we found approximately two million putative p53 BSs in human genome (ref. [13] and this study). Note that we used 70% cutoff for the PWM score, corresponding to the vast majority of the functional p53 REs (Additional File 5: Figure S3). Although some sites in our list may not be accessible to p53 in chromatin (which is known to be tissue-specific), our data indicate that the number of potential p53 BSs could be several orders of magnitude higher than the number of sites observed in ChIP assays.

### Two distinctive origins of p53 sites in Alu repeats

A significant number of the p53 BSs detected in genome-wide ChIP experiments [7] have been shown to reside in various repeat subfamilies such as ERV LTRs [14] and Alu repeats [36]. This observation posed the question on how these sites evolved - did they exist in the progenitors of the mentioned repeats, or did they arise by mutations?

By analyzing the LTR consensus sequences of the six ERV subfamilies containing p53 sites and comparing them with the subfamilies without p53 sites, Haussler and his colleagues [14] concluded that the p53 sites probably were embedded in the founders of these ERV subfamilies. On the other hand, Vingron and coworkers [36] applied the same strategy by comparing the p53 BSs with the consensus sequences of different Alu subfamilies. They found that the Alu counterparts of the p53 binding motifs contain the CG dimers in the positions corresponding to CA and TG in the p53 sites. Therefore, they suggested that the p53 sites with the CATG core motif could be generated by mutations, namely, through methylation and deamination of CpG [36].

Our analysis of the functional p53 REs showed that in two out of three locations where the p53 REs are mapped on Alu repeats (around positions 10 and 150, Boxes A/A'), the CG dimer occurs frequently in the Alu regions corresponding to the p53 core motif, implying that CG → TG mutations could give birth to p53 sites, as proposed earlier [15,36]. By contrast, in the other location (around position 85, Box B), the CG dimer is rarely found in the corresponding region of Alu sequences. Moreover, the p53 site associated with the Huntingtin gene (HTT) aligns perfectly well with the AluSq2 consensus sequence, so that there are no mismatches in the core regions (Figure 2 and Additional File 2: Figure S1), which indicates that this site probably pre-existed in the progenitors of at least some Alu subfamilies (see Box B in Figure 6). Therefore, in our opinion, the p53 sites residing in Alu repeats could be generated by both mechanisms discussed above [14,36].

### Implications for the repression of Alu transcription by p53

Previous studies showed that p53 can represses pol III transcription through interaction between the p53 N-terminal domain and TFIIIB, a factor containing TATA-binding protein [51-53]. In addition, the DNA binding domain (DBD) of p53 was also found to be critical for the transcriptional repression [54]. Based on the observation that the hot spot around position 85 is close to Box B of the pol III promoter (Figures 2 and 6), we can suggest a simple mechanism for the p53-induced repression of Alu transcription. Given that the p53 DBD is required for Alu silencing [54], it is plausible that p53 binds to this site *in vivo*; as a consequence, the N-terminal region of p53 would directly interact with TFIIIB, thereby precluding effective assembly of the pol III transcription machinery. Therefore, mutations in p53 leading to defects in DNA binding would result in pol III hyperactivity that is characteristic of many tumors [52,55]. In particular, there is direct evidence of the increased level of Alu RNA in hepatocellular carcinoma [56].

Moreover, we hypothesize that the p53 binding to Alu elements and repression of their transcription can change the expression of the host genes. This potential effect is related to the steric interaction between the pol II and pol III machineries, which, in turn, depends on the direction of Alu transcription (pol III) relative to the host gene transcription (pol II). If the two polymerases are moving in the opposite directions, one would expect that the head-on collision would slow down both of them, by analogy with the collision between the DNA replication apparatus and RNA polymerases [57]. (At least in one case [58] it is shown that pol II transcription from the upstream promoter of the human ε-globin gene is effectively blocked by pol III transcription of the Alu element from the opposite DNA strand.) If, however, the two RNA polymerases are moving co-directionally, the sterical hindrance between them is unlikely. (In principle, pol III transcription may even facilitate the pol II processing through chromatin.) Thus, upon p53 binding to Alu elements, the genes containing Alu inserts would be regulated differently. Those genes, whose transcription goes in the direction opposite to the transcription of the Alu inserts, are likely to be activated by p53. In the alternative case, when the Alu and the host gene transcriptions are co-directional, we predict either no effect upon p53 binding, or a relatively insignificant p53-induced trans-repression.

We suggest that the regulatory effect of Alu transcription on the expression of the host genes may be one of the selective factors responsible for the frequent occurrence of Alu repeats in introns of human genes.

## Conclusions

We observed that 24 out of ~160 known functional p53 REs reside in repeats and more than half of the repeat-associated REs occur in Alu elements. These REs can be further divided into two groups depending on their proximity to Boxes A/A' or Box B of the pol III promoter (Figure 1). Most of the REs overlapping with the A/A' boxes have zero length spacer. A comparison of the REs and the Alu subfamily consensus sequences showed that the CG dimers frequently occur at positions corresponding to the CA:TG dimers in the p53 core motifs, indicating that these sites could be generated by methylation and deamination of CpG, as was proposed by Zemojtel *et al*. [36].

On the other hand, the majority of the REs located close to the B box have spacer S ≠ 0. The CG dimers rarely occur in the corresponding Alu regions, suggesting that the CG → TG transition was not critical for generation of these sites. Rather, they might be present in the progenitors of at least several Alu subfamilies. Thus, we conclude that the p53 binding sites residing in Alu elements likely evolved by both of the mechanisms proposed earlier [14,36].

Analysis of potential p53 BSs embedded in Alu elements genome-wide showed that the BSs are distributed in a pattern similar to the functional REs discussed above (Figure 4). The similarity between the putative p53 BSs and the functional REs indicate that many of these BSs could be bound by p53 *in vivo*. In addition, the S spacers are distributed in a subfamily-specific manner and correlate with the Alu age: in general, the younger subfamilies are characterized by shorter spacer lengths (Table 2). It is tempting to speculate that the observed tendency could be related to the increased p53 binding affinity to its cognate binding sites in the 'younger' Alu elements. (This is because p53 binds to the sites with S = 0 stronger than to the sites with other spacer lengths [5,6].)

Note in this regard that the 'younger' Alu elements are characterized by a decreased binding affinity to proteins SRP9/14, comprising a part of the signal recognition particle (SRP) [59]. According to the hypothesis proposed by Bennett *et al*. [59], modern Alu RNAs have developed the ability to disengage from SRP9/14 more readily during reverse transcription, which, in turn, may be advantageous for Alu retrotransposition.

By analogy, here we speculate that evolution of Alu repeats (that is, several waves of Alu expansion) might be influenced by interactions with p53. We suggest that under selective pressure, the recent Alu elements mutated in such a way as to strengthen p53 binding and increase the level of the p53-induced repression of Alu transcription (see above). Keeping Alu expression below a certain level is critical both for the host and for transposable repeats, because excessive Alu transcription would be too exhaustive for the host cell (and eventually, disadvantageous for Alu as well).

Following this scenario, invasion of a 'young' Alu element (with a low level of pol III transcription) in the vicinity of a gene promoter, would not disturb pol II transcription of the gene itself. In this way, the p53 BS with a short spacer (S = 0 or 3 bp) and relatively strong p53 binding, would be easily transposed close to the transcription start site, thereby creating (or modifying) the network of p53-dependent regulation of this gene.

Finally, we wish to emphasize that the two groups of p53 REs and BSs (located close to the A/A' or B boxes in Alu repeats) are likely to have different chromatin environments, which are critical for p53 binding [10,39,46]. This observation suggests new criteria for selecting the 'strong' functional p53 sites (which may have been instrumental in evolution). In addition to the widely used various PWM-scores (measuring the level of similarity between the putative binding site and the consensus template), one can estimate the strength of a nucleosome-forming signal and the rotational positioning of the p53 site in the nucleosome fold. We anticipate that by applying this approach to genome-wide scanning of all possible p53 sites, we will be able to select only those sites which are 'properly exposed' in nucleosomes (Figure 7). One of the major problems in genome-wide analysis is the huge number of 'non-functional' p53 binding sites. We hope that by using this novel 'filtering' technique we will be able to reduce the list of potential p53 REs.

## Methods

### Identification of functional human p53 response elements occurring in repeats

The largest collection of p53 REs published recently by Riley *et al*. [6] comprises 156 sites. Note, however, that a p53 site from human hepatitis B virus (HBV) and two identical sites from the gene COL18A1 were included in this dataset. We removed the HBV site and one duplicate site of COL18A1 gene from this list. On the other hand, four p53 REs were detected experimentally in the CASP10 promoter [60], whereas only one of them was considered by Riley *et al*. [6]. Therefore, we added the other three CASP10 sites to the dataset. As a result, we obtained the list of 157 functional p53 REs, which was used in this study.

The selected p53 REs were extended on both sides by 250 bp flanking sequences extracted from human genome (NCBI Build 36). The REs and their flanks were aligned with repeats using RepeatMasker (A.F.A. Smit, R. Hubley, and P. Green, http://www.repeatmasker.org) and CENSOR [61]. The p53 REs overlapping with repeats by at least 5 bp are shown in Table 1 (see Additional File 2: Figure S1 and Additional file 3: Figure S2 for details).

### Construction of the position weight matrix to predict putative p53 binding sites

To find putative p53 BSs in human genome, we used a standard position weight matrix approach, PWM-20 [4], based on 34 experimentally validated p53 REs (Additional File 1: Tables S2 and S3). (The index 20 reflects the fact that a 'canonical' p53 BS contains two decamers RRRCWWGYYY.)

Later, the wider sets of p53 REs were collected, including a comprehensive collection of 157 sites mentioned above [6]. We decided, however, not to update our PWM-20, mostly for the two reasons. First, as the number of p53 REs increased fourfold (from 34 to 156), the number of the sites with 'broken' core motif CNNG increased about sevenfold, from 6 to 40 (data not shown). (The core motif CNNG in the center of each p53 half-site is critical for rigorous p53-DNA recognition through formation of the arginine-guanine hydrogen bonds in the major groove [62,63].) In other words, the statistical requirements imposed on the p53 sites based on the Riley's dataset [6] are more lenient (compared to PWM-20), which would greatly increase the number of 'false-positive' p53 BSs found in genome.

Second, in the previous study [4] a 70% cutoff for the PWM-20 score was determined based on the score distribution for the functional REs used to build PWM-20 (Additional File 1: Table S3). Calculation of the PWM-20 scores for the Riley's dataset [6] and for ~1,100 p53 BSs detected *in vivo *by Smeenk *et al*. [8] confirms that 70% is a 'natural' cutoff value (Additional File 5: Figure S3).

The process of generating PWM-20 score includes two stages. First, each potential site is characterized by the 'raw' score - the sum of the weight matrix elements representing occurrences of the individual bases (or dimers) at each position, see Table S2 in Additional File 1. The score is then represented in percent, 100% corresponding to the best possible BS and 0% corresponding to the worst possible BS. The cutoff value 70% (Additional File 5: Figure S3,) means that any site with PWM-20 score exceeding 70% is considered to be a putative p53 BS.

Our PWM-20 differs from the published PWM-based approaches used for p53 BS prediction [7,64] in several important aspects. First, PWM-20 is a symmetric weight matrix - that is, the score calculated for any sequence is identical to the score for its reverse complement. This requirement naturally follows from the symmetry of the DNA-bound p53 tetramer [62,63]. Second, PWM-20 utilizes dimeric frequencies for the positions 5-6 and 15-16 in the centers of decamers RRRCWWGYYY (Additional File 1: Table S2), reflecting the fact that the dimers AT and AA:TT are predominant, while the dimers TA, SW and WS are extremely under-represented in these positions (W is A or T, S is G or C). Third, PWM-20 is based entirely on the functional p53 REs (34 *in vivo *REs used for PWM-20 compared to 37 *in vitro *BSs used by Hoh *et al*. [64].)

### Collection of Alu elements in human genome

Alu elements (~1.1 million in total) were downloaded from AluGene Database [65]. The elements were separated into different subfamilies (Jo, Jb, Sx, *etc*.) based on the notations provided by the database.

## Competing interests

The authors declare that they have no competing interests.

## Authors' contributions

VBZ initiated and designed the study. FC and MVS created software and performed computations. VBZ and FC wrote the paper. All authors read and approved the final manuscript.

## Reviewers' comments

### Reviewer 1: Igor B. Rogozin (nominated by Pavel Pevzner)

The authors suggested that the primate-specific Alu repeats play an important role in shaping the p53 regulatory network in the context of chromatin. This conclusion is based on analysis of experimentally confirmed and computationally predicted p53 binding sites. The authors analyzed 160 functional p53 response elements and found that 24 of them occur in repeats. More than half of these repeat-associated response elements reside in Alu elements. This means that ~10% of experimentally verified p53 response elements are located in Alu elements. This number perfectly coincides with the fraction of Alu elements in the human genome which is ~11%. Thus there is no elevated frequency of p53 response elements in Alu elements. This may be due to a small number of experimentally confirmed p53 response elements. Unfortunately, the authors did not try to analyze recent genome-wide ChIP experiments [7,8].

***Authors' response:*** Yes, there is no elevated frequency of p53 response elements (RE) in Alu elements, but the number of well established functional p53 REs, 160, is probably too small to make final conclusions. On the other hand, the number of predicted (putative) p53 sites has a higher density than the rest of genome (see below our response to the second fragment of I.B. Rogozin's review).

As to the ChIP experiments, its analysis would not improve the above statistics, because the ChIP data provide information about the p53 binding sites (BS), both functional and non-functional.

There are several reasons why we did not analyze the ChIP DNA fragments. First, it has been done by the Haussler and Vingron groups [14,36]. Second, the available p53 ChIP fragment sets [7,8] are incomplete, with most of the detected p53 BSs positioned far away from the transcription start sites (Additional File 1: Table S1). Third, as was reported at the 15-th International p53 Workshop (October 2010, Philadelphia, USA), several groups are currently preparing more exhaustive p53 ChIP datasets, obtained for various types of normal and cancer cells.

In addition, there is a principal limitation of the ChIP technology with regard to detecting all functional p53 sites in the repeating elements such as Alu. The ChIP-seq method is based on sequencing the short 'reads' at the ends of the p53-bound DNA fragments (several hundred base pairs in length) with subsequent mapping of these 'reads' to human genome. If the unique mapping is impossible, the p53-bound DNA fragment is typically ignored. In the case of Alu repeats having a high degree of homology, the probability of occurrence of numerous identical 'reads' is rather high. As a consequence, many 'real' p53 BSs belonging to Alu repeats are missed. As far as the repeating elements are concerned, the p53 ChIP datasets will remain incomplete unless the ChIP-seq methodology is improved (e.g., the length of DNA 'reads' is increased). Therefore, in our opinion, the detailed theoretical analysis of the p53 ChIP data would be much more productive one or two years from now.

Using a position weight matrix approach, the authors found ~400,000 potential p53 binding sites in Alu elements genome-wide. Using the previous estimate (~10% of p53 RE are in Alu) we could easily calculate that the total number of potential binding sites in the human genome is ~ 4 million sites. I think that this is too generous estimate. Therefore, the vast majority of these potential binding sites are likely to be an over-prediction which is expected taking into account that the p53 consensus sequence is highly degenerate (see the Introduction). In addition, the cumulative work obtained from different laboratories suggests that p53 is not a stand-alone protein; rather, it participates in a complex network of proteins working in concert [66-68]. Therefore, even for a good binding site there is no guarantee that it will influence transcription of neighboring genes.

***Authors' response:*** We agree that the above estimate of ~4 million p53 sites is too generous. In fact, we calculated the number of p53 BSs directly: "A scan of the unmasked genome found ~2 million p53 sites with the PWM-20 scores of 70% or higher" (Results, page 8). This means that the p53 BSs are over-represented in Alu repeats (compared to the rest of genome, including the other, non-Alu repeats). See also on page 8: "the Alu elements have a (somewhat) higher 'density' of putative p53 sites compared to other repeats."

In any case, there is no doubt that some of these potential elements are functional and they might influence the transcription of neighboring genes immediately after insertion of a new copy of Alu. Although we do not know the number of such case, this possibility poses an interesting question about how primates tolerated a load of p53 binding sites in their genomes. I think that some insertions are deleterious (likely to be removed by purifying selection) and some insertions of Alu together with p53 binding sites may be utilized. This point reverberates with the classical paper of Wojciech Makalowski [69] I will use direct quotes from the abstract of this paper:

"Interspersed repetitive sequences are major components of eukaryotic genomes. ... they are often quoted as a selfish or junk DNA. Our view of the entire phenomenon of repetitive elements has to now be revised in the light of data on their biology and evolution.... I would like to argue that even if we cannot define the specific function of these elements, we still can show that they are not useless pieces of the genomes. The repetitive elements interact with the surrounding sequences and nearby genes. They may ... acquire specific cellular functions such as RNA transcription control or even become part of protein coding regions. Finally, they provide very efficient mechanism for genomic shuffling. As such, repetitive elements should be called genomic scrap yard rather than junk DNA."

The p53 binding sites nicely fit this concept taking into account that the rate of turnover of these elements is known to be high [70]. In general, transcription factor binding sites are frequently lost and gained [71]. Thus I expect that many potential binding sites have been spreading by mobile elements, however only a small fraction of these elements have been used as functional regulatory signals.

### Reviewer 2: Sandor Pongor

Tumor suppressor p53 is unique in the sense that it regulates over a thousand human genes. p53 is known to bind to a cognate site consisting of two decameric half sites separated by a spacer. Recently it has been found that many of the sites that bind p53 in vivo are within transposable elements. One should note that in vivo binding sites (BSs) are not necessarily functional p53 response elements (REs). The interesting study of Cui and associates asks the question whether or not REs also reside in transposable elements, particularly Alu repeats. The authors find that BSs are located in the same regions of Alu repeats as are REs, namely in the vicinity of A/A' and B boxes of the internal RNA polymerase III promoter. The authors conclude that Alu residing p53 sites may have evolved through a mechanism that is different from that of the corresponding Alu consensus sequences and suggest that the strongest binding sites are embedded in the young Alu repeats. My questions are:

a) Can one estimate the background probability of occurrence of the motifs tested? In other words, can one assign a statistical significance to the findings?

***Authors' response:*** To evaluate the significance of our findings, compare the peaks in Figures 3, 4 with the background. For Figure 3, the average for the peaks at S = 0, 3, 8, 14 is 182,587 (filled circles). The average and RMSD (σ) for the background are 121,135 and 9,883 respectively. (The occurrences of the spacer lengths are averaged for S varying from 1 to 13, excluding the four peaks mentioned above.) Thus, the difference between the 'peaks' and the background equals 6.22 × σ (6.22 = (182587 × 121135)/9883).

For Figure 4A, the average for the four peaks is 71,885, while the average and RMSD for the background are 10,141 and 4,234 respectively. In this case, the difference between the 'peaks' and the background equals 14.58 × σ (14.58 = (71885 - 10141)/4234). From the Student t-test http://studentsttest.com, we have p < 10^-6 ^for both cases.

b) At the first glance the periodicity of occurrence vs. spacer length on Figure 3, 4, 5 falls close to half of B-DNA periodicity that may be correlated with the sidedness of a recognition site - is the periodicity statistically insignificant?

***Authors' response:*** The peaks of occurrence corresponding to spacer S = 0, 3, 8 and 14 bp are statistically significant (see above). The differences between these S values are 3, 5 and 6 bp. Indeed, 5 and 6 are close to 10.5/2. How can one interpret this?

The Alu elements originated from the 7SL RNA, which is a part of the Signal Recognition Particle (SRP) [59]. Therefore, the occurrence of p53 sites with certain spacer S is the consequence of the 7SL RNA sequence organization: the tetramers in Box B at positions ~100 to ~110 (shown in blue, green and ochre in Figure 6), are separated by 1 and 2 bp (which corresponds to the shifts of 5 and 6 bp respectively). To find if this has any structural meaning, one has to analyze the crystal structures of the SRP, which contains 7SL RNA and several proteins [59]. We are afraid that any analysis of this kind would be based on numerous assumptions and thus would be quite speculative.

### Reviewer 3: King Jordan

#### General comments

Feng Cui *et al*. report on an analysis of Alu element derived p53 binding sites in the human genome. Transposable element (TE) derived p53 binding sites, and Alu-derived sites in particular, have been treated at some length previously both by the group of Victor Zhurkin, who originally discovered that numerous p53 binding sites are found in TE sequences, and others such as the Haussler and Vingron groups [14,36]. So presumably one challenge for the authors was to come up with a novel angle for this fairly well studied system, and this was achieved by partitioning p53 binding sites in to distinct sets for subsequent study. The authors divided p53 binding sites into three different classes, each of which differ with respect to the mode of analysis employed for their detection. First, there are p53 binding sites that are predicted based on sequence motifs. The regulatory function, and even the p53 binding affinity, of these sites are largely unknown. Second, there are p53 ChIP fragments - DNA sequence intervals experimentally characterized to be bound by p53 genome-wide. These sites are defined as being bound by p53 but their regulatory function is unknown. And finally, there are p53 response elements - DNA fragments that are bound by p53 and also experimentally characterized to have some regulatory effect, usually on a reporter gene construct. The work in this study is focused on what the authors refer to as the 'extreme sets' of p53 binding sites: ~2 million predicted p53 binding sites genome-wide, for which there is no experimental evidence of actual binding or regulatory function, and a far more restricted set of ~160 response elements with experimentally characterized binding and regulatory activities. While I can follow the logic of this dichotomy, I was never quite clear on why the ChIP DNA fragment set was not analyzed as well. In any case, this [is] an extremely interesting manuscript that should prove to be of broad interest. Accordingly, I do not hesitate to recommend publication of this work in Biology Direct. I do however have a number of comments on the analysis and interpretation of the data.

***Authors' response:*** First of all, we are sincerely grateful to I. K. Jordan for the detailed and thoughtful review. The reasons why we did not analyze the ChIP DNA fragments are given in our response to I.B. Rogozin's review (see above).

##### Major points

One of the most interesting findings in this study is based on results showing that there are two classes of p53 binding sites in Alus: a class that appears to be derived from methylation and deamination of CpG sites as previously reported by Vingron and colleagues [36] and a class of sites that appears to have been present in ancestral Alus as has been proposed for LTR-derived p53 sites by the Haussler group [14]. I am slightly confused about one observation regarding these two mechanisms. The sites derived from methylation and deamination map to the region of the element that is enriched for zero length spacers, which in turn are over-represented in young Alu elements. How is it that the youngest elements have experienced more CpG methylation and deamination than the older elements that have had many more millions of years to accumulate such mutations?

***Authors' response:*** I.K. Jordan made an interesting point by linking our observation of the progressive decrease in the length spacer (in the p53 BSs) with the existence of two different mechanisms involved in generation of p53 sites in Alu elements.

We disagree, however, with the reviewer' assessment: "The sites derived from methylation and deamination map to the region of the element that is enriched for zero length spacers" (that is, to the regions around the Boxes A/A'). Below, we show that:

(1) not all sites with S = 0 originated through CpG mutations (in the Boxes A/A');

(2) not all CpG mutations resulted in formation of the p53 sites with S = 0.

(1) To compare different Alu subfamilies in terms of their enrichment for various length spacers, in addition to the absolute numbers presented in Table 2, we need to consider the 'relative' values (similar to GC content). For example, consider the ratio (number of the p53 sites with S = 0) divided by (number of all Alu elements in the subfamily). This ratio is the highest for the Sg1 subfamily (58%) and exceeds more than twofold the ratios for the other Alu subfamilies. In other words, the AluSg1 sequences are characterized by the highest 'content' of p53 sites with S = 0. But it does not mean that the members of Sg1 subfamily experienced the highest CpG mutation rate. As follows from Figure S4-H, the Sg1 subfamily is unique in the sense that nearly all p53 sites with spacer S = 0 are mapped to the Box B, while in the other subfamilies the sites with S = 0 are mapped to the Boxes A/A' (Figures 4 and 5). The consensus AluSg1 sequence (Figure 6, Box B) suggests that most of the p53 sites in the Sg1 subfamily originated without mutations in CpG (Figure 6), and the AluSg1 elements have to be excluded from this consideration.

(2) Furthermore, mutations CG:CG-to-CA:TG are required not only for creation of the p53 sites with S = 0, but also for the sites with S = 8 and S = 14 (see consensus sequences in Figure 6, Box B). If we calculate the cumulative 'content' of p53 sites with S = 0, 8 and 14 bp (Table 2), it will be the highest for AluJo subfamily (35%). The next are the FLAM-C and AluSp subfamilies with 30%. The youngest subfamilies in Table 2, AluSc and AluY, have very low contents, 8% and 3%, respectively.

Thus, we see that the old AluJo subfamily has the highest fraction of the p53 sites which likely originated through CpG mutations.

The authors touch on a very interesting issue towards the end of the discussion, where they mention that p53 binding of Alus effectively silences their transcription by disrupting assembly of the pol III machinery. However, this discussion is curiously devoid of biological context with respect to the effects of Alu transcription on cellular function. I would urge the authors to consider speculating further on the biological significance of this idea. The dysregulation of TEs has previously been associated with a number of disorders including cancer. However, the classic models for the role of p53 in tumorgenesis are related to transcriptional activation and/or repression of host genes, and indeed much of the focus of this manuscript is on the potential effects of Alu carried p53 binding sites on host genes. But the work reported here suggests the possibility that defects in the function of p53 could lead to cancer via dysregulation (specifically upregulation) of the Alu elements themselves.

***Authors' response:*** Thank you for the suggestion - we included more biologically-related speculations in the last section of Discussion.

The authors propose a selective model whereby young Alus transpose near the promoter regions of host genes bearing p53 binding sites that disrupt the expression of the element, thereby mitigating some of its potential deleterious effects, and also can effect the regulation of the host gene. It is difficult to reconcile this model with the observation that younger Alus are enriched for the short spacer binding sites in the A-box region that appear to have evolved via CpG methylation and deamination. Thus, the intitial effect of many of these insertions would not be to bring that p53 binding site and so selection could not act at that point. This model is also inconsistent with the observation that young Alu elements are depleted near genes relative to older Alu elements.

***Authors' response:*** In our opinion, not only the young Alus bear p53 binding sites that could potentially disrupt the expression of the Alu element (through the interaction between p53 N-terminus and pol III transcriptional machinery), but the old Alu elements may also play a similar role. As shown in Figure 6, all Alu subfamilies (from FLAM to AluY) share a highly conserved p53 site in the Box A. All Alus except AluSc and AluY bear a site in the Box B with spacer S = 3, 8 or 14 bp. It is conceivable that p53 could bind these sites, disrupting the expression of Alu elements and at the same time, playing a regulatory role in the transcription of nearby host genes.

With respect to the analyses reported here, there are a few rather qualitative conclusions drawn from data that could be quantitatively and statistically analyzed in such a way as to provide more definitive results. The trends that the authors point to do seem to be there, but a more quantitative analysis could provide additional support for their conclusions. I provide a few suggestions to this end below.

First, the method for comparing the p53 binding site spacer length distributions for repetitive and nonrepetitive DNA seems indirect. It appears as if the authors compared, 1) the entire genome including repeats and non-repeats (see filled circles in Figure 3) with 2) only the non-repeat part of the genome (open circles in Figure 3). In other words, the non-repetitive fraction analysis in part 2 was done on a subset of the entire genome analysis in part 1. Why not directly compare the spacer distributions for the repeat and non-repeat parts of the genome directly?

***Authors' response:*** In Figure 3, we compared the whole genome with the non-repeat part of the genome. The latter is characterized by nearly constant frequency of occurrence of the spacer S (where S varies from 0 to 20 bp). This means that the peaks in the spacer length distribution originate (almost) entirely due to repeats. The approximate values for the repeat part of the genome can be easily obtained by subtracting 60,000 from the values presented in Figure 3 for the whole genome. (Approximate because those few p53 BSs that occur at the borders of the repeat elements would be eliminated from consideration.) In addition, the spacer length distribution for Alu repeats is given in Figure 4A.

Second, the relationship between the age of Alu elements and the length of the spacer, which has important functional implications since short spacers tend to bind p53 with higher affinity, is quite interesting. The trend the authors highlight in Table 2 does seem somewhat apparent, but this could benefit from a more definitive quantitative analysis. In particular, some of the data fit the trend of decreasing spacer length with element age, but others, such as the youngest family AluY, do not. The relative age of the families could be correlated with the average spacer length for each class, or perhaps simply the length of the most prevalent spacer, to more quantitatively evaluate the trend.

***Authors' response:*** Unfortunately, the estimates of the average age of Alu elements are contradictory in two aspects. First, there is uncertainty regarding the order of the Alu subfamilies. For example, Kapitonov & Jurka [72] proposed that the subfamily Sx is younger than Sq, whereas several other studies, including one from the Pevzner group [73], suggested that Sx is older than Sq. Second, the ages of Alu subfamilies estimated by various groups differ substantially. For instance, Kapitonov & Jurka estimated the age of AluJo to be ~80 Myr, while the corresponding estimate made by Pevzner and colleagues is ~60 Myr.

Given the noticeable discrepancy and uncertainty of the age estimate of Alu subfamilies in the literature, we preferred to present the dependence as shown in Table 2 - at the qualitative level, all is clear here. A quantitative evaluation of the correlation between the average spacer length and the age of the Alu subfamilies is hardly possible, at least for a while.

Minor points: There are a few statements that are not directly supported by the data or the literature cited.

On page 4, Haussler and co-workers [14] are cited as substantiating previous results that TEs (LTRs) contain p53 binding sites. The references cited as providing the original observations that are substantiated by Wang *et al*. [14] are both abstracts from conference proceedings; thus it is not clear what they report. Is it not the case that the Haussler paper was the first to show that TE sequences bind p53 genome-wide based on experimental evidence as opposed to simply binding site predictions? It would help to clarify this.

***Authors' response:*** Yes, it is correct that Wang *et al*. [14] were the first to show that numerous p53 binding sites detected in the p53-ChIP experiments [7] are embedded in TE sequences. We made appropriate changes in the Background, to make this clear. On the other hand, in the two short abstracts published earlier [12,13] we showed that "simply" predicted p53 binding sites reside in TEs genome-wide. Unfortunately, we were unable to publish a detailed description of our results in 2003, because at that time the idea of thousands (let alone millions) of p53 sites residing in 'junk DNA' was absolutely unacceptable in the p53 community.

On page 5, the authors mention that they find it 'remarkable' that most of the predicted Alu-derived p53 binding sites are clustered in the same regions of the elements as seen for the well characterized response elements. I don't understand why this is remarkable in light of the fact that one would expect p53 to bind Alu elements in the regions that contain consensus binding site motifs.

***Authors' response:*** In our opinion, this is "remarkable" because numerous predicted p53 BSs behave similar to those few experimentally validated p53 REs that bind to Alu repeats. This point is discussed in detail in Conclusions (third paragraph). However, to comply with the Reviewer's objection, we substituted "remarkably" by "importantly."

On page 6, the authors state that "promoter regions are enriched with Alu elements" to underscore the potential of Alu-derived p53 binding sites to influence host gene regulation and cite Polak and Domany [40] in support of this statement. In fact, this manuscript and several others show that Alus, along with other TEs, are actually substantially depleted in proximal promoter regions adjacent to transcriptional start sites (TSS) and then steadily increase in frequency moving away from the TSS. Several kb distal from the TSS Alus are indeed found in slightly higher frequencies than for the genome as a whole, and intergenic regions in particular, but this can be attributed simply to the fact that Alus are enriched in-and-around genes.

***Authors' response:*** Thank you for the correction - indeed, these are the upstream regions of the TSS (several kilobases in length) that are enriched with Alu elements, not the promoters themselves, as we wrote. We changed the end of the Background accordingly.

## Supplementary Material

Additional file 1 **Supplementary Tables S1-S3**. Click here for file

Additional file 2 **Supplementary Figure S1: Alignment of p53 functional REs with the consensus sequences of human repeats**. Click here for file

Additional file 3 **Supplementary Figure S2: Alignment of the p53 functional REs and their flanks with the consensus sequences of Alu repeats**. Click here for file

Additional file 4 **Supplementary Figure S4: Occurrence of putative p53 sites in the subfamilies FLAM-A (A), FLAM-C (B), AluJb (C), AluSq (D), AluSg (E), AluSp (F), AluSc (G), AluSg1 (H) and AluY (I)**. Click here for file

Additional file 5 **Supplementary Figure S3: PWM-20 score distributions for p53 REs and *in vivo *binding sites**. Click here for file
