# Supplementary material for: Impact of Alu repeats on the evolution of human p53 binding sites
Source: Biol Direct. 2011 Jan 6;6:2. doi: 10.1186/1745-6150-6-2 (PMC3032802; doi:10.1186/1745-6150-6-2)
Supplement: Additional file 1 — Supplementary Tables S1-S3. [file 1745-6150-6-2-S1.PDF]

SUPPLEMENTARY TABLES

Table S1 Number of p53 binding sites with the positions relative to transcription start sites (TSS)

	Total	TSS±2 kb
Cell-2006, PET-3+	325	22 (6.8%)
NAR-2008	1545	123 (8.0%)
Functional p53 REs	157	113 (72.0%)

Cell-2006, PET-3+: the data taken from Wei *et al.* [7]. The statistically significant PET-3+ set was used for analysis.

NAR-2008: the data taken from Smeenk *et al.* [8].

Functional p53 REs: the data taken from Riley *et al.* [6]. All 157 experimentally validated p53 response elements were used for analysis.

Table S2 Frequencies of mono- and di-nucleotides used in PWM-20

	1	2	3	4[a]	5-6[b]	7[a]	8	9	10	11	12	13	14[a]	15-16[b]	17[a]	18	19	20
**A**	0.426	0.324	0.493	0.000		0.000	0.044	0.066	0.096	0.338	0.199	0.382	0.000		0.000	0.096	0.081	0.088
**C**	0.154	0.066	0.029	1.000		0.000	0.500	0.669	0.419	0.147	0.066	0.074	1.000		0.000	0.382	0.529	0.331
**G**	0.331	0.529	0.382	0.000		1.000	0.074	0.066	0.147	0.419	0.669	0.500	0.000		1.000	0.029	0.066	0.154
**T**	0.088	0.081	0.096	0.000		0.000	0.382	0.199	0.338	0.096	0.066	0.044	0.000		0.000	0.493	0.324	0.426
					**5-6**									**15-16**				
					**AA**	0.287								**AA**	0.221			
					**AT**	0.390								**AT**	0.390			
					**TA**	0.029								**TA**	0.029			
					**TT**	0.221								**TT**	0.287			
					**WS**	0.009								**WS**	0.009			
					**SW**	0.009								**SW**	0.009			

[a] The cytosine in positions 4/14 and guanine in positions 7/17 are critical for the p53-DNA recognition [62,63] and conserved in the vast majority of functional p53 REs (Table S3). Therefore, any potential site having different nucleotides in these four positions is ignored by PWM-20.

[b] The dinucleotide frequencies are used for the dimers at positions 5-6 and 15-16, to reflect the fact that TA and WS:SW dimers are under-represented. (W is A or T; S is G or C). Any potential site having SS dimer at positions 5-6 or 15-16 is ignored by PWM-20.

Note: the elements of the weight matrix PWM-20 are calculated as $W(b, l) = ln\ [\ f(b, l) / p(b)\ ]$, where b is the base and l is the position in the PWM. The $f(b, l)$ are the frequencies given above, while the $p(b)$ are the frequencies of mono-nucleotides or di-nucleotides occurring in genome.
For simplicity, we define $p(A) = p(C) = p(G) = p(T) = 0.25$ and assign $p(NN) = 1/16 = 0.0625$ to each di-nucleotide.

Table S3 Functional p53 REs used to build PWM-20

#	Gene Name(s)	Spacer, bp	1st Half-site[a]	2nd Half-site[a]	Score, %
1[b]	AMID (AIFM2)	4	AGA**CATG**CCT	GCA**CTAA**TTT	N/A
1[b]	AMID (AIFM2)	1	GCA**CTAA**TTT	AAA**CAAG**CTT	N/A
2	APAF1	13	AGA**CATG**TCT	CGA**CAAG**CCC	91.3
3	BAI1	0	TGG**CT-G**CCT	GGA**CATG**TTC	N/A
4	BAX	1	TCA**CAAG**TTA	AGA**CAAG**CCT	75.0
5	BBC3	0	CTG**CAAG**TCC	TGA**CTTG**TCC	81.7
6	CASP1	0	AGA**CATG**CAT	ATG**CATG**CAC	77.4
7	CASP6	4	AGG**CAAG**GAG	AGA**CTTG**TCT	79.8
8	CCNG1	0	AGA**CCTG**CCC	GGG**CAAG**CCT	86.1
9	CCNK	2	AAA**CTAG**CTT	AGA**CATG**CTG	79.5
10	CD82	5	AGG**CAAG**CTG	GCT**CAAG**CCT	74.5
11	CDKN1A	0	GAA**CATG**TCC	CAA**CATG**TTG	84.6
12	CX3CL1	1	GGG**CATG**TTC	CAG**CTTG**TGG	76.7
13	DDIT4	0	AAA**CAAG**TCT	TTC**CTTG**ATC	70.5
14	DDR1	0	GAG**CTGG**TCC	AGG**CTTA**TCT	N/A
15	GADD45A	0	GAA**CATG**TCT	AAG**CATG**CTG	86.6
16	GDF15	0	AGC**CATG**CCC	GGG**CAAG**AAC	78.2
17	IGFBP3	1	AAA**CAAG**CCA	CAA**CATG**CTT	83.1
18	IGFBP3	0	GGG**CAAG**ACC	TGC**CAAG**CCT	77.3
19	IRF5	2	AGG**CATG**CCA	AGG**CATG**GTC	83.2
20	LRDD	8	AGG**CCTG**CCT	GGA**CATG**TCT	86.4
21[c]	MDM2 (HDM2)	0	GGT**CAAG**TTC	AGA**CACG**TTC	73.7

21[c]	MDM2 (HDM2)	0	AGTTAAGTCC	TGACTTGTCT	N/A
22	MMP2	0	AGACAAGCCT	GAACTTGTCT	93.0
23	P53AIP1	0	TCTCTTGCCC	GGGCTTGTCG	78.1
24	PMAIP1	0	AGGCTTGCCC	CGGCAAGTTG	88.0
25	PML	8	GGGCTGGCCT	GGGCATGTCC	85.6
26	PTEN	14	GAGCAAGCCC	GGGCATGCTC	93.0
27	RRM2B	0	TGACATGCCC	AGGCATGTCT	94.6
28	SERPINB5	5	GGACAAGCTG	AGGCTTGAGT	78.5
29	SFN	2	TAGCATTAGC	AGACATGTCC	N/A
30	TNFRSF10B	0	GGGCATGTCC	GGGCAAGACG	87.8
31	TP53I3	0	CAGCTTGCCC	ACCCATGCTC	76.3
32	TP53INP1	0	GAACTTGGGG	GAACATGTTT	73.9
33[b]	TP73	8	GTACTTGCCG	GAACTTGCAG	73.6
33[b]	TP73	11	GAACTTGCAG	GAGCTTGAAT	70.4
34	TRIM22	0	TGACATGTCT	AGGCATGTAG	84.3

[a] The central tetramers of the half-sites which are consistent with the consensus CNNG motif are shown in green; otherwise, the tetramers are shown in red. Note that in all the cases where both half-sites have CNNG motif, the PWM-20 score exceeds 70%.

[b] The REs (AMID and TP73) contain three decamers (D1, D2, D3) separated by spacers S1 and S2: D1 – S1 – D2 – S2 – D3.
Due to uncertainty which site to select when calculating frequencies presented in Table S2, we considered the two binding sites D1 – S1 – D2 and D2 – S2 – D3 with weight = 0.5.

[c] The RE (MDM2) contains two pairs of canonical decamers separated by 18 bp-long spacer:
D1– D2 – 18 bp – D3 – D4. In this case, two sites D1 – D2 and D3 – D4 were considered with weight = 0.5.
For references to the original papers where functionality of these p53 REs is proven experimentally, see Staib *et al.* [4].
