# Supplementary material for: Impact of Alu repeats on the evolution of human p53 binding sites
Source: Biol Direct. 2011 Jan 6;6:2. doi: 10.1186/1745-6150-6-2 (PMC3032802; doi:10.1186/1745-6150-6-2)
Supplement: Additional file 2 — Supplementary Figure S1: Alignment of p53 functional REs with the consensus sequences of human repeats. [file 1745-6150-6-2-S2.PDF]

Figure S1 Alignment of p53 functional REs with the consensus sequences of human repeats (see Table 1)

The CNNG cores in the p53 binding motifs and their ‘counterparts’ in repeats are shown in boldface and highlighted in yellow, except for the CG dinucleotides that are highlighted in magenta. Locations of the Boxes A/A’ and B in Alu repeats are shown in Figure 1. If a p53 RE is aligned with Alu repeat, its ‘div’ value is shown, which is defined as the ratio between the number of substitutions and the length of matching region.

Thirteen REs in Alu repeats

Six REs associated with Boxes A/A’ (positions 10 and 150)

AIFM2 RE2 (S = 10) div = 3/24

```
AIFM2 RE2          GGGCATGGCCAGGCACGGTGGCTCATGCCT
                         |||| ||| |||||||||| ||||
AluJo                    GGCCGGGCGCGGTGGCTCACGCCT
```

BID (S = 0) div = 5/20

```
BID                GGGCATGATGGTGCATGCCT
                   |||| || ||| ||  ||||
AluSg              GGGCGTGGTGGCGCGCGCCT
```

BNIP3L (S = 5) div = 9/25

```
BNIP3L             AAGCTAGTCTCAGTGGCGCATGCCT
                    |||  | |   |||||||  ||||
AluJo/Jr           TAGCCGGGCGTGGTGGCGCGCGCCT
```

CASP10 RE1 (S = 0) div = 5/20

```
CASP10 RE1         GGGCATGGTGGGACATGCCT
                   |||| ||||||  |  ||||
AluJo/Jr           GGGCGTGGTGGCGCGCGCCT
```

CASP10 RE2 (S = 0) div = 2/20

```
CASP10 RE2         GGGCATGGTGGCACATGCCT
                   |||| ||||||| |||||||
AluSp              GGGCGTGGTGGCGCATGCCT
```

TSC2 RE1 (S = 0) div = 4/20

```
TSC2 RE1           GGGCATGGTGGCACATGCCT
                   |||| ||||||| |  ||||
AluSg              GGGCGTGGTGGCGCGCGCCT
```

Seven REs associated with Box B (position 85)

AIFM2 RE1 (S = 8) div = 0/28

```
AIFM2 RE1           AGACCAGCCTGGGCAACATAGCGAGACC
                    ||||||||||||||||||||||||||||
AluJo               AGACCAGCCTGGGCAACATAGCGAGACC
```

BCL2L14 (S = 0) div = 3/20

```
BCL2L14             AGCCAAGGCTGGTCTTGAAC
                     ||| |||||||||| ||||
AluJr/Jo            TGCCCAGGCTGGTCTCGAAC
```

CASP6 (S = 4) div = 4/24

```
CASP6               AGGCAAGGAGTTTGAGACAAGTCT
                    |||| ||||||| ||||| || ||
AluJr               AGGCCAGGAGTTCGAGACCAGCCT
```

EPHA2 (S = 3) div = 2/23

```
EPHA2               AGACATGCCTGGCCAACATGGTG
                    ||||  |||||||||||||||||
AluSz               AGACCAGCCTGGCCAACATGGTG
```

GDF15 (S = 0) div = 4/20

```
GDF15               CATCTTGCCCAGACTTGTCT
                     || |||||||| || ||||
FLAM_C              TATGTTGCCCAGGCTGGTCT
```

HTT/HD (S = 3) div = 1/23

```
HTT                 CGCCATGTTGGCCAGGCTGGTCT
                    | |||||||||||||||||||||
AluSq2              CACCATGTTGGCCAGGCTGGTCT
```

TSC2 RE2 (S = 13) div = 6/33

```
TSC2 RE2            AGGCTAGTCTGAAACTCCTGGGCTGACGTGACC
                    ||||| ||||  |||||||||||| | |||| |
AluJb               AGGCTGGTCTCGAACTCCTGGGCTCAAGTGATC
```

Eleven REs in non-Alu repeats

CASP10 RE3 (S = 5)

```
CASP10 BS3          ATCCAAACTTGCTGG-TTTAAATCTTGGCT
                    | ||| |||  |||| ||  ||||  ||||
MIR                 AGCCAGACTGCCTGGGTTCGAATCCCGGCT
```

CCNK (S = 2)

```
CCNK                AAACTAGCTTGCAGACATGCTG
                     | ||| || ||||
MIRb                TACCTACCTCGCAG
```

COL18A1 (S = 0)

```
COL18A1             TGACATGTGTAAGCATGTAT
                    ||   |||||  |  ||| |
(TG)n               TG-TGTGTGTGTGTGTGT
```

CRYZ (S = 3)

```
CRYZ                CTGCAAGTCCATTAAACCTGTTT
                    |||  |||||||||||||| |||
THE1B               CTGTGAGTCCATTAAACCTCTTT
```

CTSD (S = 0)

```
CTSD                AACCTT-GGTTTGCAAGAGGC
                     |  || ||||| ||
MER4D               TATTTTTGGTTTACA
```

MMP2 (S = 0)

```
MMP2                AGACAAGCCTGAACTTGTCT
                    ||||| |||   || |||||
LTR88b              AGACATGCCCAGACATGTCT
```

PLK2 (S = 3)

```
PLK2                    GGTCATGATTCCTTAACTTGCCT
                        |||||     || ||||| ||||
MER34C                  GGTCACCTCCCCATAACTGGCCT
```

SCARA3 (S = 0)

```
SCARA3                  GGGCAAGCCCAGACAAGTTG
                        |||||| ||  ||| |||||
MER81                   GGGCAAACCAGGACGAGTTG
```

SCN3B (S = 0)

```
SCN3B                   TGACTT-GCTCTGCCTT-GCCT
                        || ||| ||||  |||| ||||
THE1B                   TGMCTTTGCTCCTCCTTCGCCT
```

TP53INP1 (S = 0)

```
TP53INP1                GAACTTGGGGGAACAT---------GTTT
                        |||||  ||| ||||          ||||
MER21B                  GAACTCAGGGAAACACTTTACTTACGTTT
```

TRIM22 (S = 0)

```
TRIM22                  TGACATGTCTAGGCATGTAG
                        || | || || ||||| ||
LTR10D                  TGGCGTGCCTGGGCATCTAA
```
