# Supplementary material for: Impact of Alu repeats on the evolution of human p53 binding sites
Source: Biol Direct. 2011 Jan 6;6:2. doi: 10.1186/1745-6150-6-2 (PMC3032802; doi:10.1186/1745-6150-6-2)
Supplement: Additional file 3 — Supplementary Figure S2: Alignment of the p53 functional REs and their flanks with the consensus sequences of Alu repeats. [file 1745-6150-6-2-S3.PDF]

Figure S2 Alignment of the p53 functional REs and their flanks with the consensus sequences of Alu repeats

Alu subfamily consensus sequences assigned by CENSOR are used for alignment, which was made by NCBI Blast (bl2seq).

The CNNG cores in the p53-binding motifs and their ‘counterparts’ in repeats are shown in boldface and underlined.

Thirteen REs in Alu repeats

Six REs associated with Boxes A/A’ (plus AIFM2 RE1)

AIFM2 RE1, S = 8 (#85) & AIFM2 RE2, S = 10, AluJo (#10) Identities = 219/264 (82%)

```
AIFM2  236  GGGCATGGCCAGGCACGGTGGCTCATGCCTACAGTCCCAGCACTTAGGGAGGCCAAGGCGGGAGGA  301
                  |||| ||| |||||||||| ||||  | ||||||||||| |||||||| |||||||||||
AluJo  1          GGCCGGGCGCGGTGGCTCACGCCTGTAATCCCAGCACTTTGGGAGGCCGAGGCGGGAGGA  60
            <------------RE2------------->

AIFM2  302  TCGTTTGAGCCCAGGAGTTGGAGACCAGCCTGGGCAACATAGCGAGACC-----------  350
            | | ||||||||||||||| |||||||||||||||||||||||||||||
AluJo  61   TTGCTTGAGCCCAGGAGTTCGAGACCAGCCTGGGCAACATAGCGAGACCCCGTCTCTACA  120
                                 <------------RE1----------->

AIFM2  351  -----TACAAAAATTAGCCGGGCTTGTTGGTGCACACCTGTAGTCCCAGCTTCTCGGGAA  405
                 |||||||||||||||||| || ||| || | ||||||||||||||| |||||||
AluJo  121  AAAAATACAAAAATTAGCCGGGCGTGGTGGCGCGCGCCTGTAGTCCCAGCTACTCGGGAG  180

AIFM2  406  GCTGAGGTGGGAGGATTGCTTGAGCCCAGGAGTTTGAGGCTACAGTGAACCATGATCATT  465
            |||||||  ||||||| ||||||||||||||||| |||||| |||||| | ||||||
AluJo  181  GCTGAGGCAGGAGGATCGCTTGAGCCCAGGAGTTCGAGGCTGCAGTGAGCTATGATCGCG  240

AIFM2  466  CCACTGCACTCCAGCCTAGGTGAC  489
            ||||||||||||||||| || |||
AluJo  241  CCACTGCACTCCAGCCTGGGCGAC  264
```

BID, S = 0, AluSg (#148) Identities = 257/294 (87%)

```
BID    115  GTGGCTCACGCCTGTAATCCCAGCACTTTGAGAGGCTGAGGGGGCGGATCGCAAGGTCA  174
            |||||||||||||||||||||||||||||| ||||| |||| ||||||||| | ||||||
AluSg  12   GTGGCTCACGCCTGTAATCCCAGCACTTTGGGAGGCCGAGGCGGGCGGATCACGAGGTCA  71

BID    175  GGAGTTTGAGACCCGCCTGGCCAACATAGTGAAACCCCATCTCTGCTAAAAATACAAAAA  234
            |||||| |||||| ||||||||||||| |||||||||| ||||| |||||||||||||||
AluSg  72   GGAGTTCGAGACCAGCCTGGCCAACATGGTGAAACCCCGTCTCTACTAAAAATACAAAAA  131

BID    235  TTAGCTGGGCATGATGGTGCATGCCTATAGCCCCATCTGCTTGGGGGGCTGAAGCAGGAG  294
            ||||| |||| || ||| ||  |||| ||  |||| || || ||| |||||| |||||||
AluSg  132  TTAGCCGGGCGTGGTGGCGCGCGCCTGTAATCCCAGCTACTCGGGAGGCTGAGGCAGGAG  191

BID    295  AATCACTTGAACCCAGGAGGTGGAGGTTGCAGTGAGCCGAGATTGTGCCATTGCACTCCG  354
            |||| ||||||||| ||||| |||||||||||||||||||||| | |||| ||||||||
AluSg  192  AATCGCTTGAACCCGGGAGGCGGAGGTTGCAGTGAGCCGAGATCGCGCCACTGCACTCCA  251

BID    355  GCCTGGGTAACAGAGCGAGACTCCATCTCaaaaaagaaaaaaaaaagaaagaaa  408
            |||||||  ||||||||||||||| |||||||||| |||||||||| ||| |||
AluSg  252  GCCTGGGCGACAGAGCGAGACTCCGTCTCAAAAAAAAAAAAAAAAAAAAAAAAA  305
```

BNIP3L, S = 5, AluJo (#147) Identities = 178/225 (79%)

```
BNIP3L  122  GGCTCACAACTGTAATCGCAGCACTTTGGGAGGCCGAGGTGGGAGGATTGCCTGAGGCCG  181
             |||||||  |||||||| ||||||||||||||||||||| ||||||||||| |||| ||
AluJo   14   GGCTCACGCCTGTAATCCCAGCACTTTGGGAGGCCGAGGCGGGAGGATTGCTTGAGCCCA  73

BNIP3L  182  GGAGTTTGAGCCCAGACTGGGCAACATGGTGAGACCCCCATCTCTACAAAAGCTAAAA--  239
             |||||| ||| |||| ||||||||||| | |||| |||| |||||||||||  || ||
AluJo   74   GGAGTTCGAGACCAGCCTGGGCAACATAGCGAGA-CCCCGTCTCTACAAAAAATACAAAA  132

BNIP3L  240  ---AGCTAGTCTCAGTGGCGCATGCCTGTAGTCCCTGGTA--CAGGAGGCTGAGGCAGGA  294
                |||  | |   |||||||  |||||||||||| | ||  | ||||||||||||||||
AluJo   133  ATTAGCCGGGCGTGGTGGCGCGCGCCTGTAGTCCCAGCTACTCGGGAGGCTGAGGCAGGA  192

BNIP3L  295  GGATCACAGCCT--GCCTGGGAAGTCAAGGGTGCAGTGAGCTATG  337
             |||||   || |  |||  |||  || ||| ||||||||||||||
AluJo   193  GGATC---GCTTGAGCCCAGGAGTTCGAGGCTGCAGTGAGCTATG  234
```

CASP10 RE1, S = 0, AluJo (#150) Identities = 232/278 (83%)

```
CASP10   107   GCCGTGGCTCATGCCTGTAATCCCAGCACTTTGGGAGGTTGACGTGGGAGGATTGCTTGA  166
               || |||||||| ||||||||||||||||||||||||||  || | |||||||||||||||
AluJo    9     GCGGTGGCTCACGCCTGTAATCCCAGCACTTTGGGAGGCCGAGGCGGGAGGATTGCTTGA  68

CASP10   167   GCCCAGGAGCTCGAGATCAGGCTGGGCAATGAAGTGAGAGCCAGTTTCTACAAAAACATT  226
               ||||||||| |||||| ||| ||||||||   || |||| || || ||||||||||
AluJo    69    GCCCAGGAGTTCGAGACCAGCCTGGGCAACATAGCGAGACCCCGTCTCTACAAAAA---A  125

CASP10   227   TTTAAAAATTTGCTGGGCATGGTGGGACATGCCTTTAGTTCCGGCTACTCTGGAGGTTGA  286
               |  ||||||| || |||| ||||||  |  |||| |||| || ||||||| ||||| |||
AluJo    126   TACAAAAATTAGCCGGGCGTGGTGGCGCGCCTGTAGTCCCAGCTACTCGGGAGGCTGA  185

CASP10   287   GGTGGGAGGATCACTTGAGCCCTGGAGGTTGAGGCTGCAGTGAACTATGATCTTGCCACT  346
               ||  |||||||| ||||||||| |||| | ||||||||||||| ||||||||  ||||||
AluJo    186   GGCAGGAGGATCGCTTGAGCCCAGGAGTTCGAGGCTGCAGTGAGCTATGATCGCGCCACT  245

CASP10   347   GCACTCCAGCCTGTGTGACAGAGCAAGACCCTGTCTCA  384
               ||||||||||||| | |||||||| |||||||||||||
AluJo    246   GCACTCCAGCCTGGGCGACAGAGCGAGACCCTGTCTCA  283
```

CASP10 RE2, S = 0, AluSp (#150) Identities = 254/281 (90%)

```
CASP10  103  GGCTGGGCACGGTGGCTCACACCTGTCATCTCAGCACTTTGTGAGGCCGAGGTGGGTGAA  162
             ||| |||| ||||||||||| ||||| ||| |||||||||| |||||||||| ||| | |
AluSp   1    GGCCGGGCGCGGTGGCTCACGCCTGTAATCCCAGCACTTTGGGAGGCCGAGGCGGGCGGA  60

CASP10  163  TCACCTGAGGTCGGGAGTTTGAGACCAGCCTGACCAACATGGAGAAACCCCATCTCTACT  222
             ||||||||||||||||||| ||||||||||||||||||||||||||||||| ||||||||
AluSp   61   TCACCTGAGGTCGGGAGTTCGAGACCAGCCTGACCAACATGGAGAAACCCCGTCTCTACT  120

CASP10  223  AAAAATACAAAA-TTAGCCGGGCATGGTGGCACATGCCTGTAATCCCAGATACTCAGGAG  281
             |||||||||||| |||||||||| ||||||| ||||||||||||||||| ||||| ||||
AluSp   121  AAAAATACAAAAATTAGCCGGGCGTGGTGGCGCATGCCTGTAATCCCAGCTACTCGGGAG  180

CASP10  282  GCTGAGGCAGGAGAATCACTTGAACCCGAGAGGCAGAGGTTGCAGTGAGCCGAGATCATG  341
             ||||||||||||||||| |||||||||| ||||| |||||||| |||||||||||||  |
AluSp   181  GCTGAGGCAGGAGAATCGCTTGAACCCGGGAGGCGGAGGTTGCGGTGAGCCGAGATCGCG  240

CASP10  342  CCATTGCACTGCAGCCTGCATAACAAGAGCGAAACTCCATC  382
             |||||||||| |||||||   ||||||||||||||||| ||
AluSp   241  CCATTGCACTCCAGCCTGGGCAACAAGAGCGAAACTCCGTC  281
```

TSC2 RE1, S = 0, AluSg (#148) Identities = 243/277 (87%)

```
TSC2    112  GGGTGCGGGGGCTTATGCCTGTAATCCTAGGACTTTGGGAGGCCGAGGTGGGTGGATCAC  171
             ||| |||| |||| | ||||||||||| || ||||||||||||||||| ||| |||||||
AluSg   5    GGGCGCGGTGGCTCACGCCTGTAATCCCAGCACTTTGGGAGGCCGAGGCGGGCGGATCAC  64


TSC2    172  GAGGTCAGGAGTTCAAGACCACTCTGGCCAACATGGCGAAACCCCGTCTGTACTAAAAG-  230
             |||||||||||||| ||||||  ||||||||||||| |||||||||||| ||||||||
AluSg   65   GAGGTCAGGAGTTCGAGACCAGCCTGGCCAACATGGTGAAACCCCGTCTCTACTAAAAAT  124


TSC2    231  ---ATGATGAGCCGGGCATGGTGGCACATGCCTGTAATCCCAGCTACTTGGGAGGCTGAG  287
                |  || |||||||| ||||||| |  ||||||||||||||||||| |||||||||||
AluSg   125  ACAAAAATTAGCCGGGCGTGGTGGCGCGCGCCTGTAATCCCAGCTACTCGGGAGGCTGAG  184


TSC2    288  GCAGGAGAATCGCTTGAACCCGGGAGGCACAGGTTGCAGTGAGCCGAGATCGCCCCACTG  347
             ||||||||||||||||||||||||||||  ||||||||||||||||||||||| ||||||
AluSg   185  GCAGGAGAATCGCTTGAACCCGGGAGGCGGAGGTTGCAGTGAGCCGAGATCGCGCCACTG  244


TSC2    348  CACTCCAGTCTGGGTGACGGAGCAAGACTCTGTCTCa  384
             |||||||| ||||| ||| |||| |||||| ||||||
AluSg   245  CACTCCAGCCTGGGCGACAGAGCGAGACTCCGTCTCA  281
```

(In the TSC2 231–287 line, the two CATG motifs are printed bold and underlined; in the AluSg 125–184 line, CGTG and CGCG are printed bold and underlined.)

Six REs associated with Box B (AIFM2 RE1 is shown above, together with AIFM2 RE2)

BCL2L14, S = 0, AluJr (#87) Identities = 260/310 (83%)

(NOTE: Alu is in the reverse orientation)

```
BCL2L14  39   tttttttttttctttatttttGTTTCTTAAGAGAGTGTCTCCCTCTGCCACCCAGGCTGG  98
              ||||||||||| ||| ||||| ||| || ||| || ||||| ||||| | ||||||||||
AluJr    310  TTTTTTTTTTTTTTTTTTTTTTTTTTTTGAGACAGGGTCTCGCTCTGTCGCCCAGGCTGG  251


BCL2L14  99   AGTGCACTGGCGCGATCATAGCTCACTGCAGCCTCCAACTCCCAGGCTCAAGCGATTCTC  158
              |||||| |||||||||||||||||||||||||||| ||||||  |||||||||||| |||
AluJr    250  AGTGCAGTGGCGCGATCATAGCTCACTGCAGCCTCGAACTCCTGGGCTCAAGCGATCCTC  191


BCL2L14  159  CCCCCTCAGCCTCCCAAGTGGCTGGGACTGCAGGTGCACACCACCACGCCCAGCTAAttt  218
              || |||||||||||| ||| ||||||||| |||| || | ||||||||||| ||||||||
AluJr    190  CCGCCTCAGCCTCCCGAGTAGCTGGGACTACAGGCGCGCGCCACCACGCCCGGCTAATTT  131


BCL2L14  219  ttttcattttttGTAGAGACGG-------------AGCCAAGGCTGGTCTTGAACTCCTG  265
              || | |||||||||||||||||              ||| |||||||||| |||||||||
AluJr    130  TTAT-ATTTTTTGTAGAGACGGGGTCTCGCTATGTTGCCCAGGCTGGTCTCGAACTCCTG  72


BCL2L14  266  GGCTTAAGCGATCCTGCTCCCTGGGCCTCCCAAAGTGCTGGGATTACAGGCGTGAGCCAC  325
              | || |||||||||| |  ||| |||||||||||||||||||||||||||||||||||||
AluJr    71   GCCTCAAGCGATCCTCCCGCCTCGGCCTCCCAAAGTGCTGGGATTACAGGCGTGAGCCAC  12


BCL2L14  326  TGGGTCCGGC  335
               | | |||||
AluJr    11   CGCGCCCGGC  2
```

CASP6, S = 4, AluJr (#80) Identities = 253/304 (83%)

```
CASP6   172   GGCCAGGTGTGGTGGTTCATGCTGGTAATCCCAGCACTTTGGGAAGCCAAGGTGGGAGGA  231
              |||| || | ||||| ||| ||  |||||||||||||||||||| ||| ||| |||||||
AluJr   1     GGCCGGGCGCGGTGGCTCACGCCTGTAATCCCAGCACTTTGGGAGGCCGAGGCGGGAGGA  60

CASP6   232   TCACTTAAGGCAAGGAGTTTGAGACAAGTCTGGGCAACAGAGTGAGACCTGGTCTCTACa  291
              || ||| |||| ||||||| ||||| || |||||||||| || ||||||  |||||||||
AluJr   61    TCGCTTGAGGCCAGGAGTTCGAGACCAGCCTGGGCAACATAGCGAGACCCCGTCTCTACA  120

CASP6   292   aaaaataaaaaTCAATTAGCTGGGCATGGTGGCG-------GTAGTCCCAGCTACTCTGG  344
              ||||||| |||  ||||||| |||| ||||||||       |||||||||||||||| ||
AluJr   121   AAAAATATAAA--AATTAGCCGGGCGTGGTGGCGCGCGCCTGTAGTCCCAGCTACTCGGG  178

CASP6   345   AGGCTGAAGGGGGAGGATTGCTTGATCCCAGGAGTTCAAGGCTGCACGGAGCTGTGATCA  404
              ||||||| | |||||||| |||||| ||||||||||| ||||||||  ||||| |||||
AluJr   179   AGGCTGAGGCGGGAGGATCGCTTGAGCCCAGGAGTTCGAGGCTGCAGTGAGCTATGATCG  238

CASP6   405   TGCCACTGCACTCCAGGCTGGGCAACAGAGTGAGACCTTGTCTCtaaaataaaaaataaa  464
               ||||||||||||||| |||||| |||||| |||||| |||||| |||| |||||| |||
AluJr   239   CGCCACTGCACTCCAGCCTGGGCGACAGAGCGAGACCCTGTCTCAAAAAAAAAAAAAAAA  298

CASP6   465   ataa  468
              | ||
AluJr   299   AAAA  302
```

EphA2, S = 3, AluSz (#93) Identities = 246/285 (86%)

```
EphA2  163   GGGCAGGGTGGCTCAGGCCTGTAATTCCAGCACTTTGGGAGGCCGAGGCAGGCAGATCAT  222
             ||||  ||||||||| ||||||||| ||||||||||||||||||||||| ||| |||||
AluSz  5     GGGCGCGGTGGCTCACGCCTGTAATCCCAGCACTTTGGGAGGCCGAGGCGGGCGGATCAC  64

EphA2  223   TTGAGGTCAGGAGCTCCAGACATGCCTGGCCAACATGGTGAAACCCCGTCTCTACTAAAA  282
             ||||||||||||| || ||||  |||||||||||||||||||||||||||||||||||||
AluSz  65    TTGAGGTCAGGAGTTCGAGACCAGCCTGGCCAACATGGTGAAACCCCGTCTCTACTAAAA  124

EphA2  283   ATACaaaaaaaTTAGCTGGGCGTGGTGCCACTCACCTGTAATCCCATCTACTAAGGAGGC  342
             ||||  |||||||||| |||||||||| | | | |||||||||||| |||||  ||||||
AluSz  125   ATAC--AAAAATTAGCCGGGCGTGGTGGCGCGCGCCTGTAATCCCAGCTACTCGGGAGGC  182

EphA2  343   TAAGGCAGGAGAATCACTTGAACCCCAGAGGCAGAGATTGCAGTGAGCCGAGATTGCGCC  402
             | ||||||||||||| |||||||||  ||||| ||| ||||||||||||||||| ||||
AluSz  183   TGAGGCAGGAGAATCGCTTGAACCCGGGAGGCGGAGGTTGCAGTGAGCCGAGATCGCGC-  241

EphA2  403   ACTCACTGCACTCCAGCCTGGGC----CAGCAAGACTCTGTCTCa  443
                ||||||||||||||||||||     ||| |||||| ||||||
AluSz  242   ---CACTGCACTCCAGCCTGGGCGACAGAGCGAGACTCCGTCTCA  283
```

In the EphA2 223–282 line, CATG (twice) is bold and underlined; in the AluSz 65–124 line, CCAG and CATG are bold and underlined.

GDF15, S = 0, FLAM_C (#92) Identities = 106/127 (83%)

(NOTE: FLAM_C is in the reverse orientation)

```
GDF15    214   TTCttttttttAGAGATGAGGTATTGCCATCTTGCCCAGACTTGTCTCGAACTCCTGGGC  273
               || ||||||||||||| | ||| | || || |||||||| || |||||||||||||||||
FLAM_C   128   TTTTTTTTTTTAGAGACGGGGTCTCGCTATGTTGCCCAGGCTGGTCTCGAACTCCTGGGC  69


GDF15    274   TCAAACAATCCACCCACCTCGGCCTCCCAAAGTGCTGAGATTACTGACATAAGCCACCAT  333
               |||| | |||| ||| ||||||||||||||||||||| |||||| | | | |||||||
FLAM_C   68    TCAAGCGATCCTCCCGCCTCGGCCTCCCAAAGTGCTGGGATTACAGGCGTGAGCCACCGC  9


GDF15    334   GCCTGGC  340
               ||| |||
FLAM_C   8     GCCCGGC  2
```

HTT(HD), S = 3, AluSq2 (#93) Identities = 266/299 (88%)

(NOTE: Alu is in the reverse orientation)

```
HTT     43   tttatttatttatttatttttGAGACAGAGTCTCACTCTTGTCACCCAGGCTGGAGTGCA  102
             ||| ||| ||| ||| |||||||||| |||| || |||||||| ||||||||||||||||
AluSq2  304  TTTTTTTTTTTTTTTTTTTTTGAGACGGAGTTTCGCTCTTGTCGCCCAGGCTGGAGTGCA  245

HTT     103  ATGGCATGATCTTGGCTCACTGCAACCTCCACCTCCCAGGTTCAAGCAATTCT---GCCT  159
             |||||  ||||| ||||||||||||||||| |||||| ||||||||| |||||   ||||
AluSq2  244  ATGGCGCGATCTCGGCTCACTGCAACCTCCGCCTCCCGGGTTCAAGCGATTCTCCTGCCT  185

HTT     160  CAGCCTCCGGAATAGCTGGGACTACAGGCATGCACCACTACACCCGGCTAATTTTTGTAT  219
             |||||||| || ||||||||| |||||||   | |||| || ||||||||||||||||||
AluSq2  184  CAGCCTCCCGAGTAGCTGGGATTACAGGCGCCCGCCACCACGCCCGGCTAATTTTTGTAT  125

HTT     220  TTTTAGTAGAGACAGGGTTTCGCCATGTTGGCCAGGCTGGTCTCGAACTCCTGACCTCTG  279
             ||||||||||||| ||||||| |||||||||||||||||||||||||||||||||||| |
AluSq2  124  TTTTAGTAGAGACGGGGTTTCACCATGTTGGCCAGGCTGGTCTCGAACTCCTGACCTCAG  65

HTT     280  GTGATCTGCCTGCCTTGGCCTCCCAAAGTGCTGGGATTACAGGCGTGAGCCACCGCACC  338
             |||||| ||| |||| |||||||||||||||||||||||||||||||||||||||| ||
AluSq2  64   GTGATCCGCCCGCCTCGGCCTCCCAAAGTGCTGGGATTACAGGCGTGAGCCACCGCGCC  6
```

TSC2 RE2, S = 13, AluJb (#75) Identities = 234/282 (82%)

(NOTE: Alu is in the reverse orientation)

```
TSC2    44    GAGACAAGGTCTCACTCTGTCACCCAGGCCGGGGTGCAGTGTCACAATCATGGCTCACTA  103
              |||||| |||||| ||||||| ||||||| || |||||||| | | |||| ||||||||
AluJb   282   GAGACAGGGTCTCGCTCTGTCGCCCAGGCTGGAGTGCAGTGGCGCGATCACGGCTCACTG  223

TSC2    104   CAGCCTCAACCTGCCAGGCTCACGTGATCCTCCTACTTTAGCCTCTCAAGTAGCTGGGAC  163
              ||||||| |||| || |||||| | ||||||||| | | |||||| | ||||||||||||
AluJb   222   CAGCCTCGACCTCCCGGGCTCAAGCGATCCTCCTGCCTCAGCCTCCCGAGTAGCTGGGAC  163

TSC2    164   TACAGGCATGCAGCACCACGCCTGGCTAAGTTTTGTATTTTTTGTCGTGACAGGGTCTCA  223
              |||||||  ||  ||||||||| |||||| ||||||||||||||| | ||| |||| |||
AluJb   162   TACAGGCGCGCGCCACCACGCCCGGCTAATTTTTGTATTTTTTGTAGAGACGGGGTTTCA  103

TSC2    224   CTGTGTTGCCCAGGCTAGTCTGAAACTCCTGGGCTGACGTGACCCTCTCCCCTCTGCCAC  283
              |  ||||||||||||| ||||  |||||||||||| | |||| |||| | |||| ||| |
AluJb   102   CCATGTTGCCCAGGCTGGTCTCGAACTCCTGGGCTCAAGTGATCCTCCCGCCTCGGCCTC  43

TSC2    284   CCGAAGTGCTGAGATTACAGGTGTGAGCCACCGCACTTGGCC  325
              || |||||||| ||||||||| |||||||||||| |  ||||
AluJb   42    CCAAAGTGCTGGGATTACAGGCGTGAGCCACCGCGCCCGGCC  1
```
