# Supplementary material for: Impact of Alu repeats on the evolution of human p53 binding sites
Source: Biol Direct. 2011 Jan 6;6:2. doi: 10.1186/1745-6150-6-2 (PMC3032802; doi:10.1186/1745-6150-6-2)
Supplement: Additional file 4 — Supplementary Figure S4: Occurrence of putative p53 sites in the subfamilies FLAM-A (A), FLAM-C (B), AluJb (C), AluSq (D), AluSg (E), AluSp (F), AluSc (G), AluSg1 (H) and AluY (I). [file 1745-6150-6-2-S4.PDF]

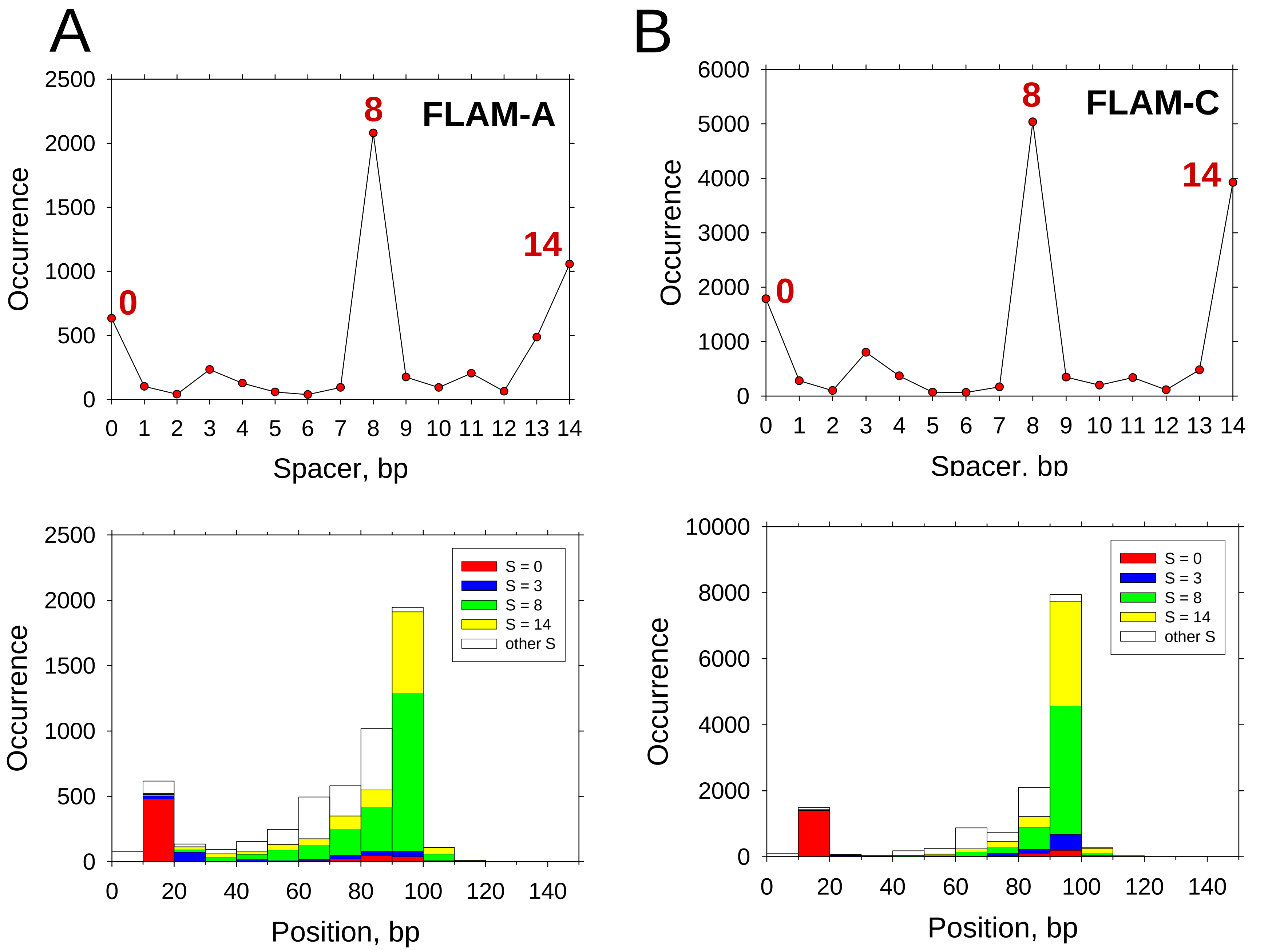

A
B
FLAM-A
FLAM-C
Occurrence
Spacer, bp
Position, bp
8
14
0
S = 0
S = 3
S = 8
S = 14
other S

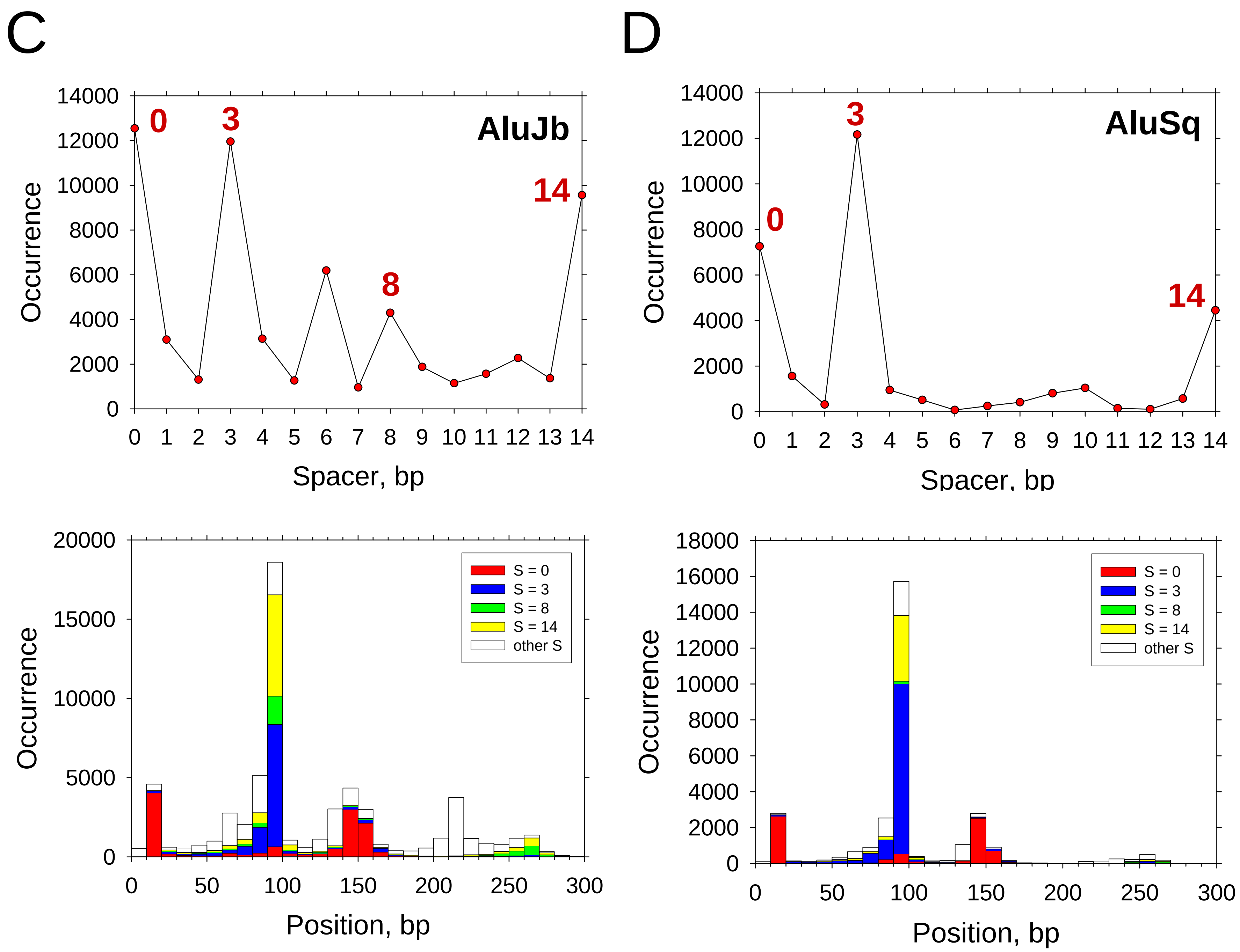

C
D
AluJb
AluSq
0
3
8
14
Occurrence
Spacer, bp
Position, bp
S = 0
S = 3
S = 8
S = 14
other S

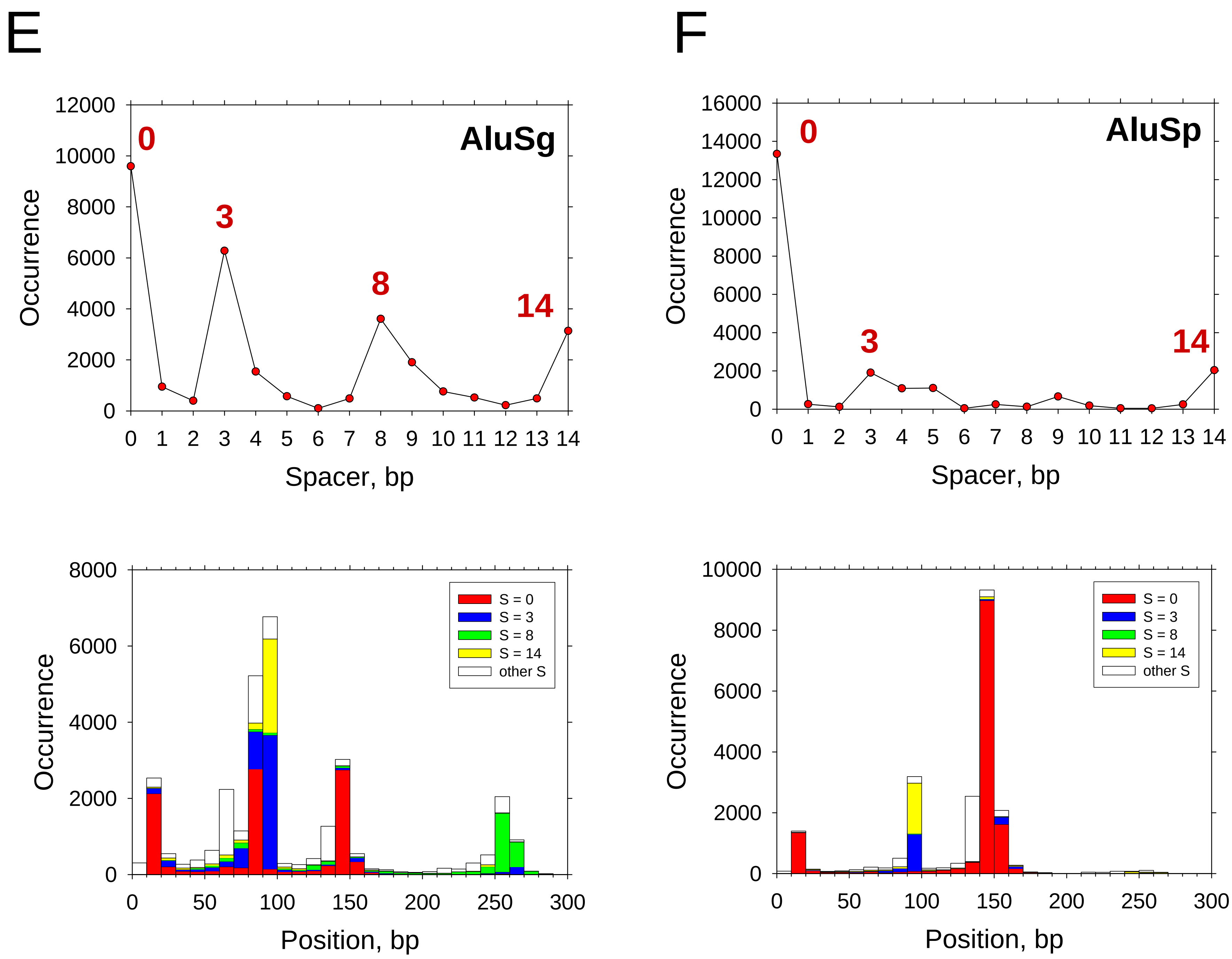
E
F
AluSg
AluSp
0
3
8
14
Occurrence
Spacer, bp
Position, bp
S = 0
S = 3
S = 8
S = 14
other S

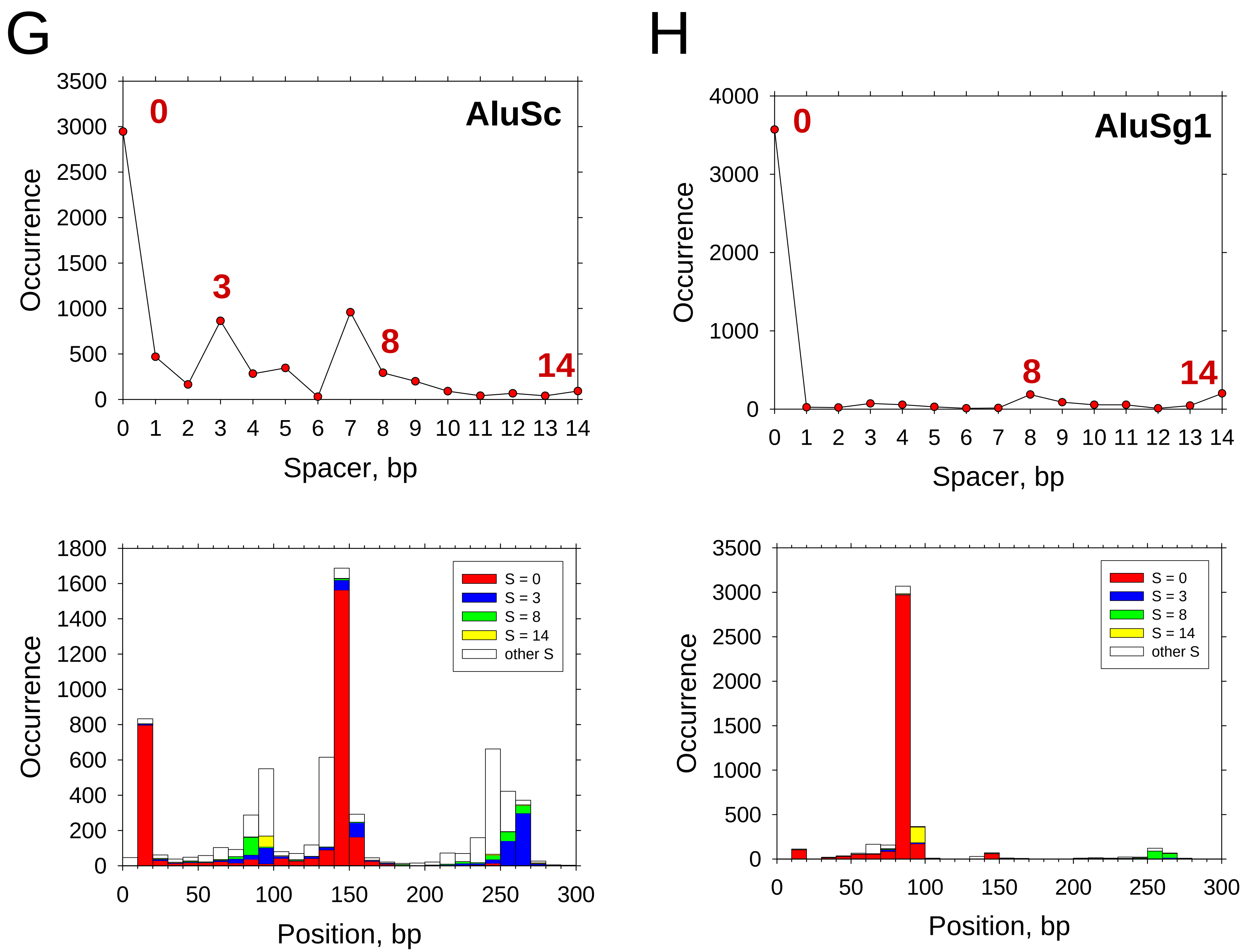
G
AluSc
Occurrence
Spacer, bp
0
3
8
14
0 1 2 3 4 5 6 7 8 9 10 11 12 13 14
0 500 1000 1500 2000 2500 3000 3500
Occurrence
Position, bp
0 50 100 150 200 250 300
0 200 400 600 800 1000 1200 1400 1600 1800
S = 0
S = 3
S = 8
S = 14
other S
H
AluSg1
Occurrence
Spacer, bp
0
8
14
0 1 2 3 4 5 6 7 8 9 10 11 12 13 14
0 1000 2000 3000 4000
Occurrence
Position, bp
0 50 100 150 200 250 300
0 500 1000 1500 2000 2500 3000 3500
S = 0
S = 3
S = 8
S = 14
other S

I

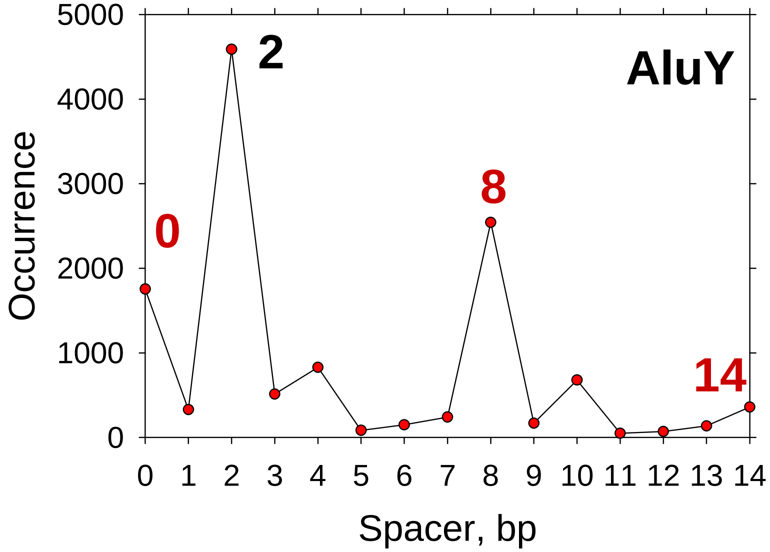

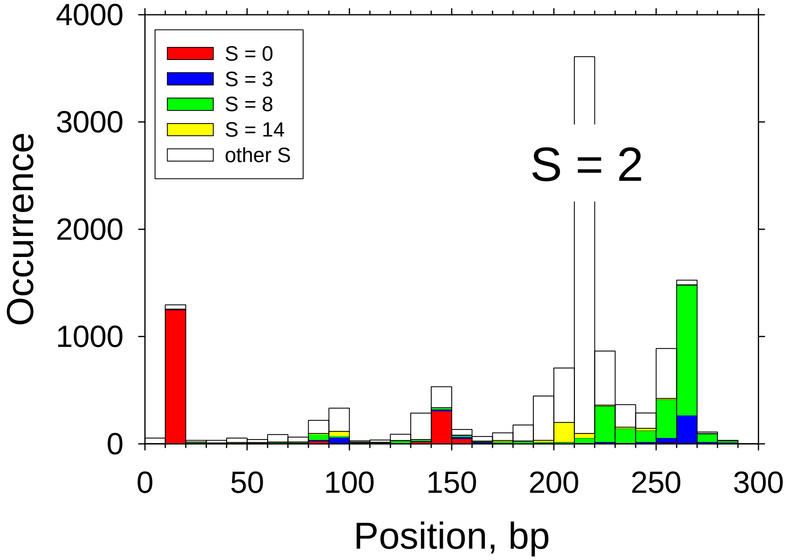

Figure S4 Occurrence of putative p53 sites in the subfamilies FLAM-A (A), FLAM-C (B), AluJb (C), AluSq (D), AluSg (E), AluSp (F), AluSc (G), AluSg1 (H) and AluY (I)

The notations are the same as in Figure 4. Note that the AluSg (E) and AluSg1 (H) are the only subfamilies where a significant fraction of the p53 sites mapped to the Box B (position ~85) have spacer S = 0.
