# Supplementary material for: Impact of Alu repeats on the evolution of human p53 binding sites
Source: Biol Direct. 2011 Jan 6;6:2. doi: 10.1186/1745-6150-6-2 (PMC3032802; doi:10.1186/1745-6150-6-2)
Supplement: Additional file 5 — Supplementary Figure S3: PWM-20 score distributions for p53 REs and in vivo binding sites. [file 1745-6150-6-2-S5.PDF]

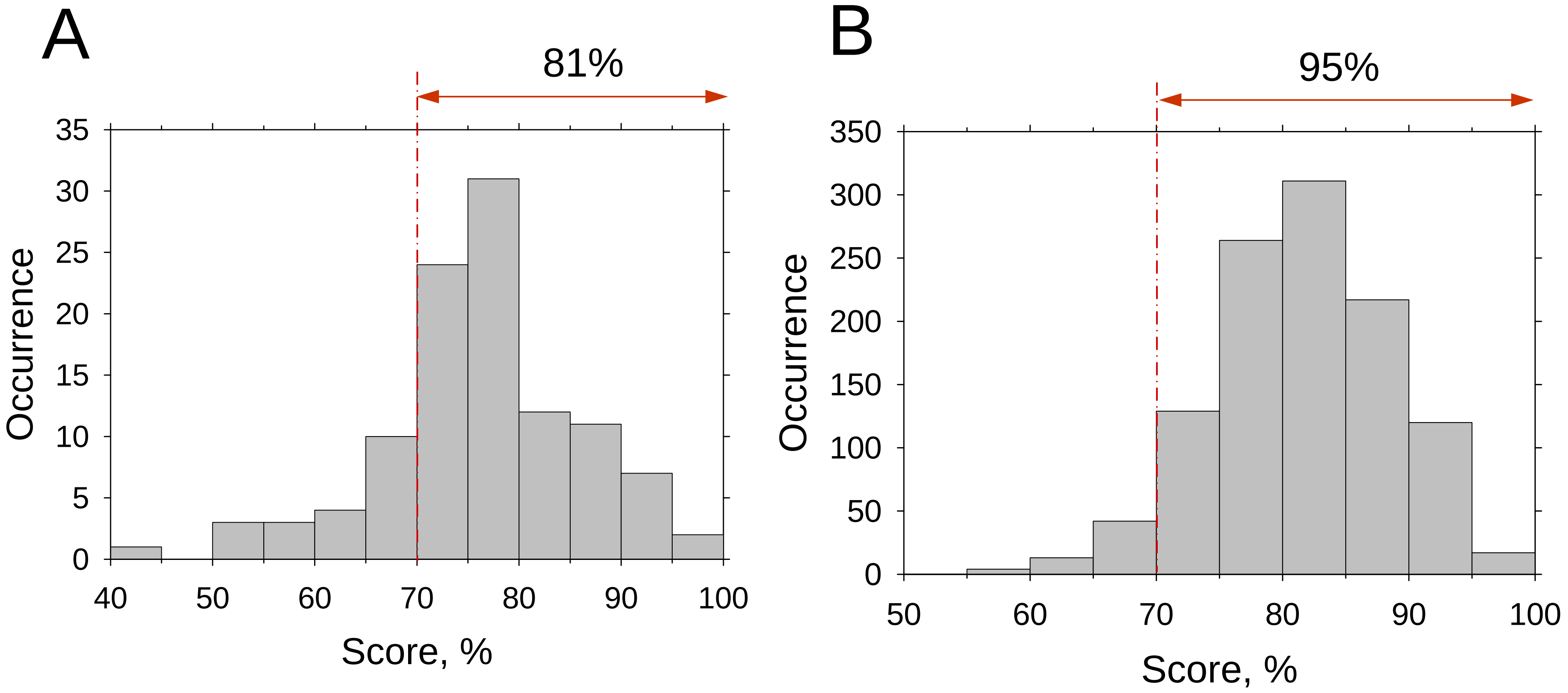

Figure S3 PWM-20 score distributions for p53 REs and *in vivo* binding sites

(A) Distribution of PWM-20 scores for p53 REs. Out of the 157 REs published by Riley *et al.* [6], 44 do not have canonical CWNG:CNWG motifs in the centers of the decamers, and two REs have spacer S > 14 bp; these 46 REs were not assigned any score (see Methods). The histogram shows distribution of the PWM-20 scores for the remaining 108 REs.
Note that 81% of these REs have PWM-20 scores higher than 70% cutoff (shown by red line).
(B) Distribution of PWM-20 scores for the p53 BSs detected *in vivo* by Smeenk *et al.* [8]. Using chromatin immunoprecipitation, the authors localized 1281 p53 BSs, 1117 of which are close to the p53 consensus and satisfy the criteria presented in Methods.
The PWM-20 scores were calculated for these sites, and in 95% of them (1058 of 1117), the score exceeded 70% cutoff.
